# Validation and high-resolution downscaling of Sentinel-2 biophysical variables using PlanetScope in vineyard and annual cropping systems

**DOI:** 10.3389/fpls.2026.1974500

**Published:** 2026-10-30

**Authors:** César Minuesa, Jordi Gené-Mola, Èlia Capdevila, Joaquim Bellvert

**Affiliations:** IRTA, Efficient Use of Water in Agriculture, Fruitcentre, Lleida, Catalonia, Spain

**Keywords:** biophysical variables, data fusion, machine learning, PlanetScope, precision agriculture, Sentinel-2 SL2P, vineyard modelling

## Abstract

Accurate estimation of vegetation biophysical variables, including the fraction of absorbed photosynthetically active radiation (fAPAR), fraction of vegetation cover (FVC), and leaf area index (LAI), is essential for crop monitoring and management. Although Sentinel-2 provides operational biophysical variable products at 20 m resolution, uncertainties remain across crop types, particularly in woody systems such as vineyards, and this resolution is often insufficient to capture within-field variability. This study aimed to (i) validate the Sentinel-2 Simplified Level-2 Prototype (SL2P) product over Mediterranean herbaceous and vineyard crops; and (ii) develop and assess a PlanetScope–Sentinel-2 fusion framework to generate 3-m, high-frequency maps of fAPAR, FVC, and LAI. SL2P showed strong agreement with *in-situ* reference data for all variables (fAPAR: R^2^ = 0.88, |bias| = 0.02, uncertainty = 0.07; FVC: R^2^ = 0.80, |bias| = 0.08, uncertainty = 0.09; LAI: R^2^ = 0.86, |bias| = 0.48, uncertainty = 0.62), with fAPAR showing the lowest absolute bias. To increase spatial and temporal detail while preserving physical consistency, four downscaling approaches were compared. DMS-DT provided a favorable compromise between predictive performance and computational efficiency (fAPAR: R^2^ = 0.73, |bias| = 0.03, uncertainty = 0.10; FVC: R^2^ = 0.67, |bias| = 0.11, uncertainty = 0.13; LAI: R^2^ = 0.75, |bias| = 0.52, uncertainty = 0.75), with residual correction improving performance. Overall, the proposed fusion framework enables 3-m, high-frequency monitoring of biophysical variables while maintaining consistency with Sentinel-2 SL2P products. The resulting products could provide a potentially useful data source for applications requiring fine-scale and frequent vegetation information, including irrigation management, yield prediction, and other precision-agriculture applications.

## 1 Introduction

The estimation of vegetation biophysical variables—notably the fraction of absorbed photosynthetically active radiation (fAPAR), fraction of vegetation cover (FVC), and leaf area index (LAI)—is fundamental for agricultural monitoring, water and nutrient management, and crop modeling. Satellite-derived products provide spatially continuous and temporally consistent information on crop status, supporting yield prediction, irrigation scheduling, orchard management, and decision-support systems (Bellvert et al., 2023; Geraud et al., 2026; Li et al., 2025b; Quintanilla-Albornoz et al., 2025). In crop modeling applications, however, the spatial and temporal resolution, as well as the uncertainty of these variables, directly influence model parameterization, evaluation, and predictive performance. Operational applications therefore require both sufficient spatial detail to capture within-field heterogeneity and frequent observations to characterize rapid canopy changes, a combination that is rarely achieved by a single sensor.

Field measurements—destructive LAI sampling, ceptometer- or hemispherical-photography-based fAPAR and LAI estimates, and image-based FVC observations—remain essential for calibration and validation, yet their spatial and temporal coverage is limited (Jonckheere et al., 2004). UAVs and satellite platforms increasingly fill this gap by upscaling *in-situ* data: UAVs resolve sub-plot heterogeneity, while satellites offer regional coverage. Two main retrieval strategies are commonly used. Empirical models relate vegetation indices or spectral features to field measurements and often perform well within the calibration domain but have limited transferability and are prone to saturation (Brown et al., 2025; Chen et al., 2025; Gao et al., 2022; Minuesa et al., 2025). Physics-based approaches instead invert radiative transfer models and are generally more transferable but computationally more demanding and sensitive to model assumptions (Li et al., 2026; Myneni et al., 2015; Peng et al., 2024).

The Sentinel-2 Simplified Level-2 Prototype (SL2P) generates operational LAI, fAPAR, and FVC products at 20 m resolution and 5-day revisit using a hybrid approach that combines radiative transfer model inversion and machine learning (Weiss et al., 2020). SL2P has become a widely used source of biophysical information for vegetation monitoring, with generally good performance in herbaceous crops, although underestimation has been reported in dense canopies and its performance in structurally complex woody crops such as vineyards remains less well established (Brown et al., 2021; Djamai et al., 2019; Fernandes et al., 2023, 2014; Hu et al., 2020; Putzenlechner et al., 2019). Yet even Sentinel-2 cannot deliver the spatial, temporal, and spectral characteristics needed for continuous field-scale monitoring simultaneously. The PlanetScope constellation, by contrast, is designed to provide frequent observations at roughly 3 m resolution—substantially finer in space and time—and its SuperDove generation adds improved radiometric performance and eight spectral bands (Kabir et al., 2025; Ma et al., 2025; Tu et al., 2022). Its narrower spectral sampling, however—only one red-edge band and no SWIR coverage—limits the direct application of radiative transfer models such as PROSAIL relative to spectrally richer sensors like Sentinel-2 (Fernandes et al., 2024; Houborg and McCabe, 2018a). Sentinel-2 and PlanetScope are thus complementary: Sentinel-2 supplies the spectral information needed for robust biophysical retrieval, while PlanetScope contributes the spatial and temporal detail required for fine-scale, frequent monitoring.

Data fusion has emerged to exploit the complementary strengths of different satellite sensors and offset their individual spatial, temporal, and spectral limitations. Classical approaches include spatial sharpening methods, such as TsHARP, which exploit empirical relationships between fine-resolution predictors and coarser-resolution target variables (Agam et al., 2007; Kustas et al., 2003), and spatiotemporal fusion methods, such as STARFM and its extension, which combine the spatial detail of high-resolution sensors with the temporal frequency of coarser-resolution observations (Gao et al., 2006; Hilker et al., 2009; Zhu et al., 2016). Effective as they are, these methods typically rely on predefined or simple relationships that struggle to capture the nonlinear interactions of heterogeneous agricultural landscapes (Merlin et al., 2010; Wei et al., 2019). The growing availability of multisource remote-sensing data has therefore driven the adoption of machine-learning methods—regression trees, random forests, and artificial neural networks—capable of learning more complex patterns directly from the data (Chen and Zhang, 2023; Main-Knorn et al., 2026; Zhang et al., 2026). Data Mining Sharpener (DMS) methods exemplify this shift, applying data-mining algorithms to link coarse-resolution targets with fine-resolution spectral information (Gao et al., 2012; Guzinski et al., 2023). More recently, deep-learning architectures—convolutional neural networks (CNNs), long short-term memory networks (LSTMs), and Transformers—have gained traction for spatial and spatiotemporal fusion (M. Li et al., 2025a; Liu et al., 2026; Michel and Inglada, 2026; Tsardanidis et al., 2025), though they generally demand larger training datasets and greater computational resources.

PlanetScope has likewise been folded into data-fusion frameworks to compensate for its limited spectral sampling. Earlier studies combined it with spectrally richer sensors to synthesize missing bands and sharpen the spatial or temporal resolution of vegetation indices (Amankulova et al., 2024; Kong et al., 2023; Main-Knorn et al., 2026; Sarkar et al., 2024). Two strategies dominate biophysical variable retrieval from PlanetScope: direct estimation from its reflectance via radiative transfer models and machine learning, and downscaling of biophysical variables derived from spectrally richer sensors (Johansen et al., 2022). took the first route, generating daily CubeSat-based LAI estimates in maize with an RMSE of 0.73 m^2^ m^-2^, though LAI values above 5 were underestimated and validation relied on 7 × 7-pixel windows. Dong et al. (2024) applied a model-driven approach based on LESS to SuperDove imagery for GLAI retrieval in wheat, reaching an RMSE of 0.80 m^2^ m^-2^. The second route was followed by Houborg and McCabe (2018a) and Sadeh et al. (2021), who fused PlanetScope with Landsat 8 and Sentinel-2, respectively, to estimate LAI, reporting relative mean absolute differences of 4–6% in alfalfa and an uncertainty of 1.73 m^2^ m^-2^ in wheat. Both studies, however, relied on earlier PlanetScope sensors, and the Sadeh et al. (2021) used temporally interpolated Sentinel-2 inputs rather than direct frequent observations.

In this work, it is hypothesized that sharpening and data-mining methods can be adapted to combine PlanetScope imagery with SL2P-derived fAPAR, FVC, and LAI to generate high-frequency 3 m products, although lower retrieval bias is expected in vineyards than in herbaceous crops, particularly for LAI, due to the structural complexity of woody canopies. Accordingly, the study aims to (i) validate Sentinel-2 SL2P biophysical variables in herbaceous crops and vineyards, and (ii) compare, and validate fused Sentinel-2/PlanetScope fAPAR, FVC, and LAI products across representative Mediterranean cropping systems, including maize, winter cereals, alfalfa, and vineyards with and without inter-row cover crops.

## 2 Materials and methods

### 2.1 Data acquisition & pre-processing

#### 2.1.1 Study area

The study was conducted in irrigated plots of the Ebro Basin, northeastern Spain (Catalonia), under a Mediterranean climate (**Figure 1**). The dataset comprised 33 plots: 13 winter cereal, 9 alfalfa, 9 maize, and 2 vineyards (Raimat and Aranyó). Each vineyard was split into two management sub-zones: conventional tillage (Vines+tillage) and cover cropping (Vines+covercrop). Although both vineyards used cover cropping, conditions diverged sharply. In Raimat, a shallow water table sustained vigorous cover-crop growth throughout the season, requiring three mowing events, whereas in Aranyó the cover crop established poorly and senesced early. Canopy architecture also differed between sites: Raimat used a trellis system with vertical shoot positioning, while Aranyó retained a traditional bush-vine system. These contrasts were expected to influence the remote-sensing signal.

**Figure 1:**
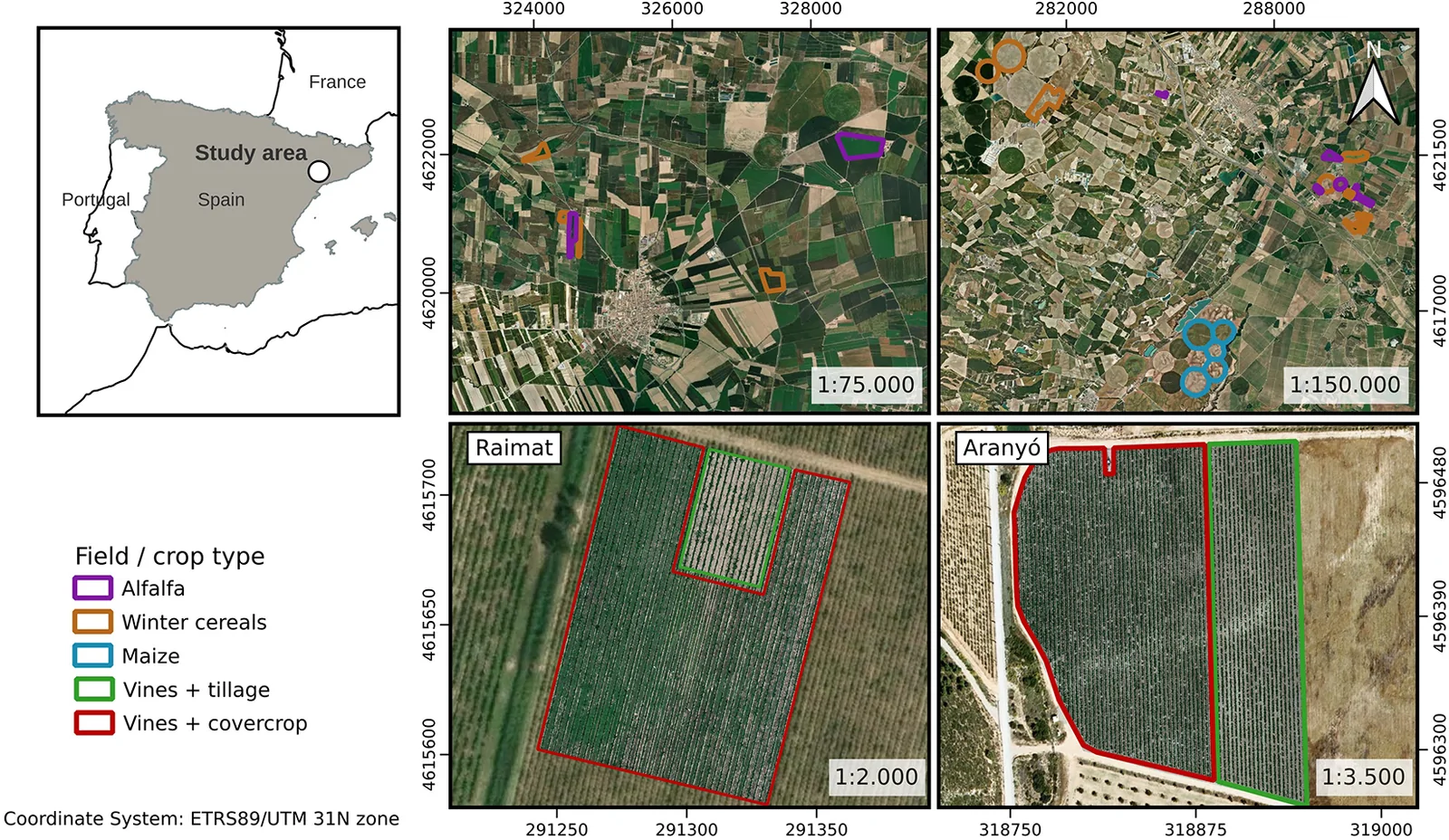
The four locations of the study area – plots sampled by crop type, and the reference names are indicated on the vineyard trials.

#### 2.1.2 Field measurements and UAV-derived reference data

The fAPAR, FVC, and LAI were measured *in situ* using ground sampling units of 0.25 m^2^ (0.5 × 0.5 m) for herbaceous crops and vineyard cover crops. In vineyards, each sample corresponded to a complete vine matching row spacing (4.25 m^2^ in Raimat, 3.9 m^2^ in Aranyó).

Field *fAPAR* measurements were estimated from the photosynthetically active radiation balance using a ceptometer (AccuPAR LP80, Decagon Devices Inc., Pullman, WA, USA), following the simplified formulation of Baret et al. (1989) (Equation 1).

(1) $$fAPAR=R_{\infty }\cdot \left(1-T_{C}\right)$$

Canopy transmittance (*T_C_*) was derived from above- and below-canopy PAR readings taken around 10:45 local time under stable illumination: two above- and three below-canopy readings for herbaceous and cover crops, and fifteen below-canopy readings spanning the full vine sampling unit for vineyards, to capture row structure and within-canopy heterogeneity. The asymptotic canopy reflectance for complete absorption (*R*_∞_) was fixed at 0.94 (Baret et al., 1989) and verified against measured canopy reflectance (*R_C_*) in closed canopies where FVC ≈ 1 (Appendix A. *fAPAR & LAI calibration*).

Field *FVC* measurements were obtained from image-based methods adapted to crop structure. For herbaceous crops, RGB images were used to compute the Normalized Green-Red Difference Index (NGRDI), and pixels with NGRDI >0.60 were classified as crop. For vineyards, UAV multispectral imagery and a vegetation height model, computed as the difference between the digital surface model and digital terrain model, were combined. Pixels with SAVI > 0.21 and vegetation height > 0.60 m were classified as vines, while pixels with SAVI > 0.21 and vegetation height ≤ 0.60 m as cover crop Appendix B. *Vineyard UAV-derived reference data*.

*In situ LAI* measurements were derived from an indirect ceptometer-based approach using canopy transmittance (*T_C_*) and the Norman–Jarvis radiative transfer model (Norman and Jarvis, 1974). To correct for saturation and clumping effects, these estimates were calibrated against destructive LAI measurements obtained with a leaf area meter (LI-3100, LI-COR Inc., Lincoln, NE, USA). Crop-specific calibration functions were then applied to all measurements Appendix A. *fAPAR & LAI calibration*.

Given the spatial uniformity of herbaceous crops, three samples were averaged per Sentinel-2 pixel (20 × 20 m) and one per PlanetScope pixel (3 × 3 m). Vineyards, by contrast, show pronounced within- and between-row heterogeneity that sparse point sampling rarely captures (Weiss et al., 2004). To address this spatial heterogeneity, UAV surveys were used to generate high-resolution fAPAR and LAI maps for vines and cover crops. Empirical models following Gao et al. (2022) and Minuesa et al. (2025), were calibrated using the UAV observations and the corresponding field measurements, and subsequently applied to generate spatially continuous UAV-derived reference maps at the UAV spatial resolution. These maps were aggregated to the corresponding satellite footprints and used as spatial reference data for satellite-product validation. Thus, unlike the direct field measurements described above, the UAV-derived reference maps represent model-based estimates and include uncertainty associated with the empirical modeling step. To limit point-spread-function and adjacency effects, only central pixels within each plot were retained for evaluation, applying the same central-pixel filtering to the UAV-derived reference maps. Full preprocessing and calibration details for vineyard fAPAR, FVC, and LAI reference data are given in Appendix B. *Vineyard UAV-derived reference data*. Hereafter, reference data refers to the values used to validate each satellite pixel: field measurements aggregated to the satellite footprint in herbaceous crops, and UAV-derived reference maps in vineyards.

Reference data was paired with satellite imagery within a 3-day window, retaining only pixels on slopes under 6% (Fernandes et al., 2024). The 33 independent field locations satisfy the CEOS Stage 2 validation criteria [>30 sites with intercomparison against similar products; (Fernandes et al., 2014)].

#### 2.1.3 Satellite data

##### 2.1.3.1 Sentinel-2 imagery

*Sentinel-2* imagery for all four study sites was obtained from the Copernicus Data Space Ecosystem throughout 2024. The Sentinel-2 constellation (Sentinel-2A and Sentinel-2B) carries the MultiSpectral Instrument (MSI), which acquires 13 spectral bands across the visible, near-infrared (NIR), and shortwave infrared (SWIR) regions at 10, 20, and 60 m spatial resolution, with ground sampling distance matching pixel resolution for each band (Drusch et al., 2012). All scenes were acquired around 10:45 a.m. local solar time.

Level-1C Top-Of-Atmosphere products were converted to Level-2A Bottom-Of-Atmosphere reflectance using Sen2Cor (v2.12) in SNAP (v9.0), and cloud- and shadow-affected pixels were masked using the scene classification layer.

##### 2.1.3.2 PlanetScope imagery

PlanetScope imagery was obtained from the Planet Labs archive via the Planet API; only SuperDove acquisitions from 2024 were used. SuperDove sensors acquire eight narrow spectral bands—six visible, one red-edge, one NIR—at a native ground sampling distance of 3.7–4.2 m, resampled to 3 m.

All images were PlanetScope Level-3B Surface Reflectance products (orthorectified and atmospherically corrected), harmonized to Sentinel-2 as the target sensor to improve radiometric consistency across PlanetScope acquisitions and with Sentinel-2. The harmonization framework draws on multi-temporal, multi-sensor reference sampling to limit inconsistencies from atmospheric effects, calibration differences, and BRDF effects (Houborg and McCabe, 2018b). It does not, however, apply an explicit physical BRDF correction or normalize to a common viewing geometry. Residual directional reflectance effects may therefore persist, particularly over row-structured vineyards. The provided cloud masks have known limitations (Fitts et al., 2025). All scenes were therefore manually inspected, and only cloud-free imagery acquired around 11:00 local solar time was retained. This matched Sentinel-2 acquisition conditions and minimized differences in solar illumination geometry.

### 2.2 Methodology pipeline

#### 2.2.1 Retrievals of biophysical variables at 20 m using SL2P

SL2P, implemented in SNAP, was used to derive fAPAR, FVC, and LAI from Sentinel-2 Level-2A Bottom-Of-Atmosphere reflectance (Weiss et al., 2020). It relies on back-propagation artificial neural networks trained on a large synthetic dataset generated with the PROSAIL radiative transfer model (Jacquemoud et al., 2009). PROSAIL couples the PROSPECT leaf optical model, which simulates leaf reflectance and transmittance from biochemical and structural properties, with the SAIL canopy model, which accounts for canopy architecture, leaf angle distribution, hotspot effects, and soil background.

Through this inversion framework, SL2P simultaneously estimates fAPAR, FVC, LAI, and albedo from Top-Of-Canopy reflectance. The LAI product corresponds to effective green LAI, accounting for clumping effects and including all green vegetation elements observed by the sensor. fAPAR is given as an instantaneous value under clear-sky conditions at approximately 10:45 local solar time—closely approximating the daily integrated value—and covers only green vegetation elements. FVC gives the fraction of green vegetation cover as seen from nadir.

Hereafter, the SL2P-derived product using Sentinel-2 data will be denoted as *fAPAR_SL_*_2_*_P_*, *FVC_SL_*_2_*_P_* and *LAI_SL_*_2_*_P_*.

#### 2.2.2 PlanetScope–Sentinel-2 fusion for spatio-temporal downscaling

A spatio-temporal PlanetScope–Sentinel-2 fusion methodology was developed to exploit the improved radiometric consistency and spectral resolution of the latest PlanetScope generation, SuperDove. The overall workflow is shown in **Figure 2**.

**Figure 2:**
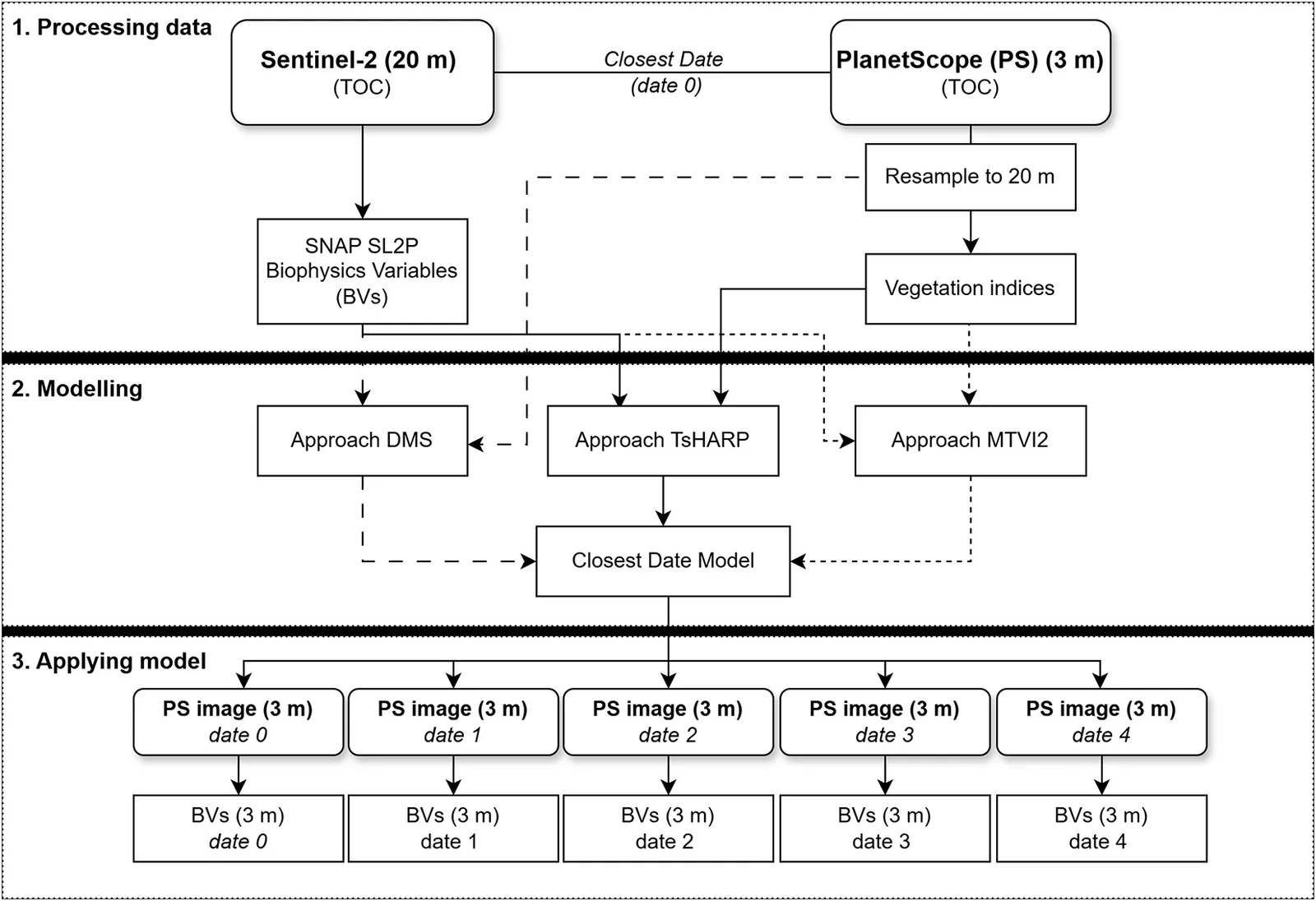
Workflow of downscaling Sentinel-2 (20 m) to PlanetScope (3 m) daily biophysical imagery using the MTVI2, TsHARP and Data Mining Sharpener (DMS) methods. Models were calibrated using the closest Sentinel-2–PlanetScope pair (date 0) and subsequently applied to consecutive PlanetScope acquisitions until a new matching pair became available, at which point the models were recalibrated.

Each cloud-free Sentinel-2 SL2P acquisition (date 0) was paired with the temporally closest PlanetScope image, keeping only pairs separated by ≤3 days (Fernandes et al., 2024); most pairs differed by ≤1 day given PlanetScope’s high revisit frequency. The downscaling methods were trained over a ~1 km^2^ calibration area surrounding each study site, chosen to ensure sufficient spatial variability. Within this area, Sentinel-2 SL2P biophysical variables at 20 m acted as targets, and PlanetScope surface reflectance and vegetation indices—aggregated to the same 20 m grid—acted as predictors, using only pixels clear of clouds and shadows in both sensors. The trained model was then applied to subsequent PlanetScope images to generate high-resolution products until the next Sentinel-2–PlanetScope match became available, at which point it was recalibrated to account for seasonal changes in canopy structure, illumination, and local reflectance relationships.

(i) MTVI2 linear regression. A univariate linear regression using the Modified Triangular Vegetation Index 2 (MTVI2) as the sole predictor. Training samples were generated by collocating Sentinel-2 SL2P biophysical variables at 20 m with PlanetScope reflectance aggregated to 20 m, from which MTVI2 was computed. MTVI2 was chosen following Sadeh et al. (2021), who identified it as the most effective index. This simple, transparent method serves as a baseline but cannot capture multivariate or strongly non-linear relationships.

(ii) TsHARP-style method. PlanetScope NDVI was converted into a fractional cover proxy (Agam et al., 2007). Linear regressions between this proxy and each SL2P biophysical variable were fitted from near-concurrent PlanetScope–Sentinel-2 pairs, then applied to the 3 m proxy data to produce downscaled fAPAR, FVC, and LAI maps.

(iii–iv) Data Mining Sharpener (DMS). The DMS framework of Gao et al. (2012), with refinements from Guzinski et al. (2023), was applied using two non-linear algorithms: (iii) an artificial neural network (DMS-ANN) with one to three hidden layers (50–100 neurons, ReLU activation, Adam optimizer), and (iv) a bagged ensemble of decision trees (DMS-DT) parameterized as in Gao et al. (2012). In both cases, 80% of samples were used for training and 20% for validation, with an independent reference dataset (Section 2.1.2) reserved for testing.

All downscaling methods were further refined in three ways. First, training samples were weighted by homogeneity: all pixels were retained, but the most heterogeneous 20% were down-weighted. Second, local (moving-window) and global regressions were computed and combined, giving greater weight to local models to account for the spatial complexity of irrigated areas. Third, residual correction was applied to remove the differences between the predicted 3 m maps and the Sentinel-2 SL2P reference. For each PlanetScope date with a concurrent Sentinel-2 acquisition, the predicted 3 m map was resampled (averaged) to the 20 m Sentinel-2 grid, and the residual was calculated as the pixel-wise difference between the SL2P product and the resampled prediction. This 20 m residual was then added uniformly to all 3 m pixels within each Sentinel-2 pixel, so that the corrected map, when aggregated to 20 m, reproduces the SL2P product. Only the concurrent SL2P product is used in this step; no field or UAV data are involved. For PlanetScope dates without a concurrent Sentinel-2 acquisition, the residual map from the closest date with a Sentinel-2 match was applied in the same way to correct the predicted maps and reduce box-like artifacts.

Hereafter, the fused PlanetScope–Sentinel-2 SL2P products are denoted as $fAPAR_{Planet-SL2P\;fusion}$, $FVC_{Planet-SL2P\;fusion}$ and $LAI_{Planet-SL2P\;fusion}$.

#### 2.2.3 Statistical analysis

Model performance was evaluated following good validation practices (Fernandes et al., 2023) for biophysical products using the coefficient of determination (R^2^), bias (B), precision (P), uncertainty (U, equivalent to RMSE) and the uncertainty agreement ratio (UAR) for each biophysical variable using Equations 2–5.

(2) $$B=\;\frac{1}{N}\sum_{i=1}^{N}\left({\hat{y}}_{i}-y_{i}\right)$$

(3) $$P=\;\sqrt{\frac{1}{N}\sum_{i=1}^{N}{\left({\hat{y}}_{i}-y_{i}-A\right)}^{2}}$$

(4) $$U=\;\sqrt{\frac{1}{N}\sum_{i=1}^{N}{\left({\hat{y}}_{i}-y_{i}\right)}^{2}}$$

(5) $$UAR=\;\frac{1}{N}\sum_{i=1}^{N}I\left(\left|\frac{{\hat{y}}_{i}-y_{i}}{y_{i}}\right|\leq \;\epsilon_{rel}\cup \;\left|{\hat{y}}_{i}-y_{i}\right|\;\leq \;\epsilon_{\text{max}}\right)$$

where for each of the *N* comparisons, ${\hat{y}}_{i}$ denotes the estimated biophysical variable and *y_i_* the corresponding reference measurement (Section 2.1.2). Bias (B) represents the signed difference between the estimated and reference values, whereas absolute bias (|B|, |bias|) represents its magnitude irrespective of direction. The parameters *ϵ_rel_* and *ϵ*_max_ represent the relative and maximum target uncertainty requirements, respectively, and *I* the indicator function, as defined by Global Climate Observing System (GCOS, 2024).

## 3 Results

### 3.1 Validation of Sentinel-2 SL2P biophysical retrievals

**Figure 3** compares Sentinel-2 SL2P estimates of fAPAR, FVC, and LAI with field measurements and UAV-derived reference data for herbaceous crops and vineyards. Overall, SL2P agreed well with the reference data, supporting its use in the subsequent fusion and downscaling analyses. Note that, for vineyards, the reference data are UAV-derived model estimates rather than direct field observations, so the reported statistics include the uncertainty of the reference maps (Appendix B. *Vineyard UAV-derived reference data*).

**Figure 3:**
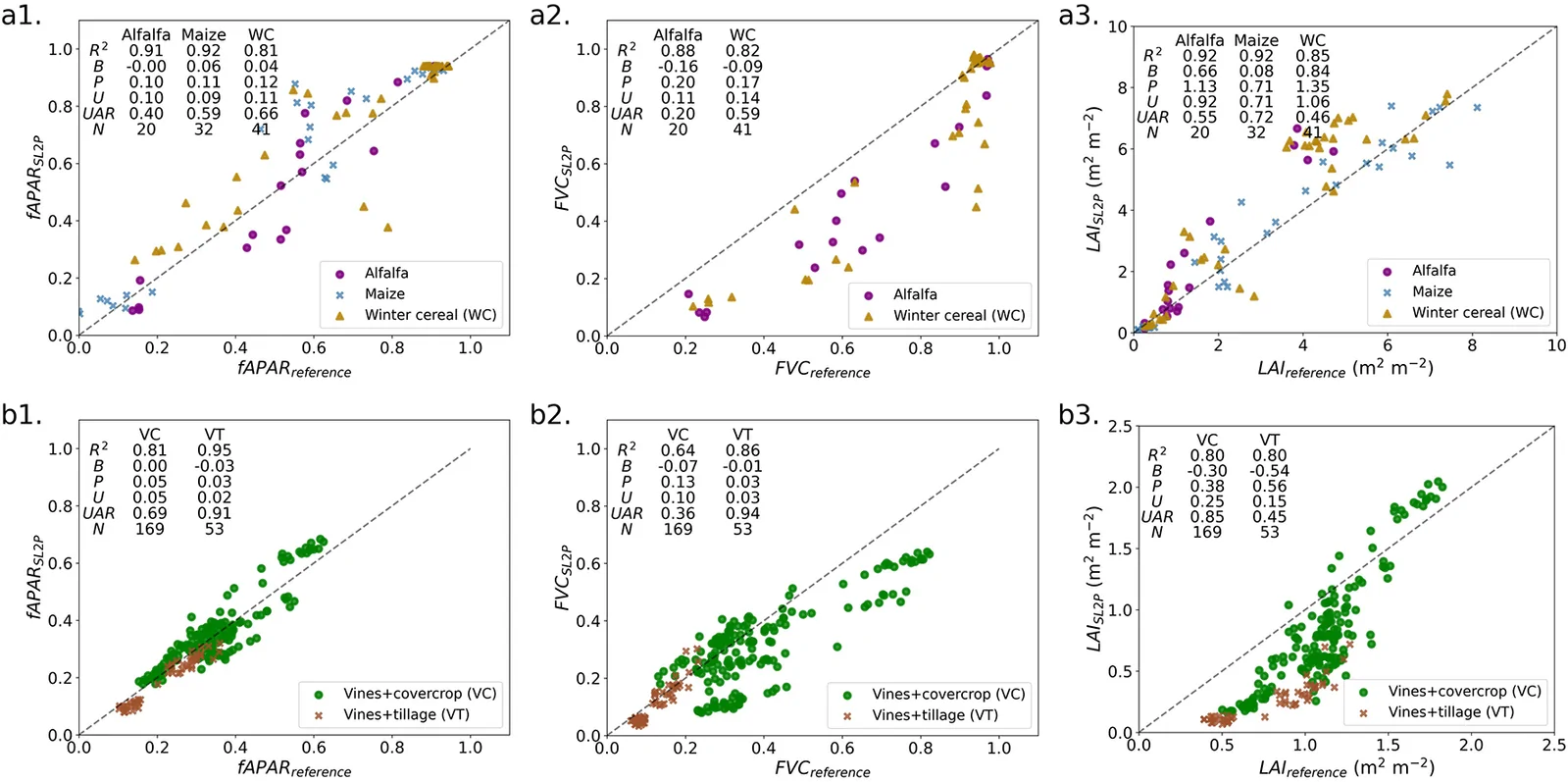
Pixel-level scatterplot validation of Sentinel-2 SL2P-derived biophysical parameters against reference dataset, showing (a1) fAPAR, (a2) FVC, and (a3) LAI for herbaceous crops, and (b1) fAPAR, (b2) FVC, and (b3) LAI for vineyards. The dashed line indicates the 1:1 agreement.

*fAPAR* retrievals correlated strongly with the reference data across all crops, with R^2^ ranging from 0.81 in winter cereals to 0.95 in tilled vineyards, while absolute bias and uncertainty stayed low throughout (|bias|< 0.06; uncertainty< 0.11). Performance was similar or better in vineyards than in herbaceous crops, especially under tillage, where residual variability was minimal.

*FVC* likewise agreed moderately to highly with the reference data, with R^2^ of 0.82–0.88 in herbaceous crops and tilled vineyards, dropping to 0.64 under cover-crop vineyards. Despite this drop, absolute bias and uncertainty remained bounded (|bias|< 0.16; uncertainty< 0.14). Tilled vineyards gave the best overall performance, while the lower absolute bias under cover crops likely reflects greater sub-pixel heterogeneity within the 20 m Sentinel-2 footprint.

*LAI* was also well correlated with the reference data (R^2^ of 0.85–0.92 for herbaceous crops, 0.80 for vineyards), but showed larger errors than fAPAR and FVC, consistent with its wider dynamic range and greater sensitivity to canopy structure. Maize gave the most reliable LAI estimates, while alfalfa and winter cereals showed larger errors. In vineyards, LAI was systematically underestimated, particularly under tillage. Uncertainty remained low under both vineyard conditions, although the bias was more negative under tillage (bias = −0.54 m^2^ m^-2^) than cover crops (bias = −0.30 m^2^ m^-2^), which may indicate a lower influence of clumping-related effects under cover-crop conditions. **Figure 4** further shows that LAI error increased with vine LAI, suggesting that canopy structural effects may contribute to the remaining error. Seasonal trajectories further indicate that SL2P captures the main LAI dynamics, although uncertainty increases at peak canopy development.

**Figure 4:**
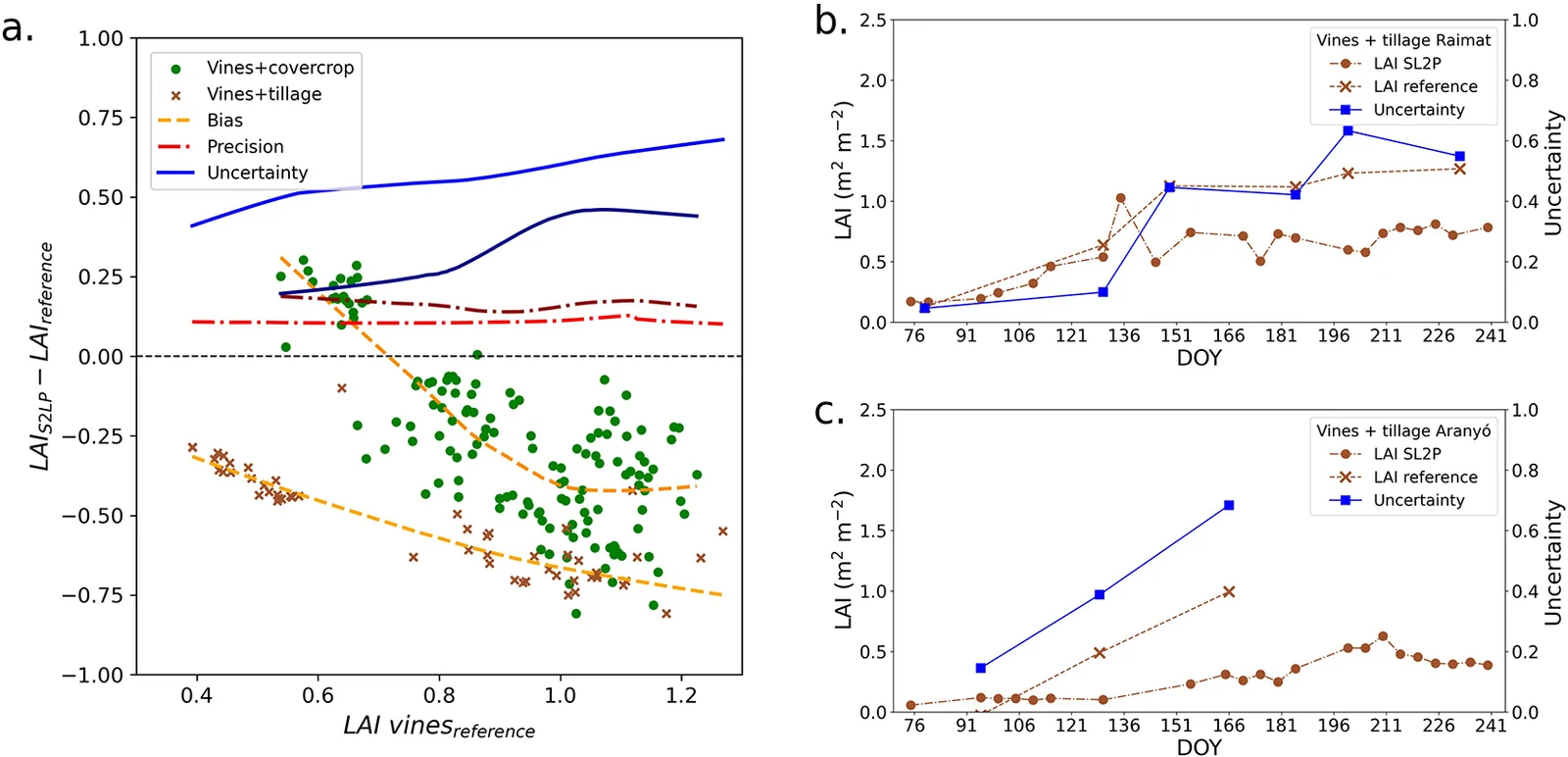
**(a)** Residuals of SL2P-derived LAI relative to UAV-derived LAI for vineyards under soil tillage and cover crop, with fitted bias, precision, and uncertainty curves. **(b, c)** Seasonal trajectories of SL2P and UAV-derived LAI, including uncertainty envelopes, for the Raimat **(b)** and Aranyó **(c)** vineyards under soil tillage management.

### 3.2 Validation of PlanetScope–Sentinel-2 fused biophysical variable emulation

#### 3.2.1 Spatial validation

To assess spatial performance, the four downscaling methods were first compared against Sentinel-2 SL2P products before residual correction, to quantify how accurately each reproduced the reference product used for training. Their actual emulation performance was then evaluated against reference data acquired on the same dates.

Across all variables, the data-mining sharpening methods (DMS-DT/DMS-ANN) outperformed the vegetation-index-based methods (MTVI2/TsHARP) (**Figure 5**). For fAPAR, MTVI2/TsHARP reached R^2^ of 0.95–0.96 and uncertainty of 0.06, while DMS methods raised performance to R^2^ of 0.98–0.99 and uncertainty of 0.03. FVC followed a similar pattern. The contrast was sharpest for LAI, where MTVI2/TsHARP showed larger errors (uncertainty = 0.75–0.79 m^2^ m^-2^; R^2^ = 0.79–0.84), while DMS-DT/DMS-ANN markedly improved agreement (uncertainty< 0.23 m^2^ m^-2^; R^2^ ≈ 0.98). Vegetation-index methods further tended to overestimate intermediate LAI values and underestimate high ones (**Figures 5a, b**), a pattern also observed across the other study areas (**Supplementary Figures S1-S5**).

**Figure 5:**
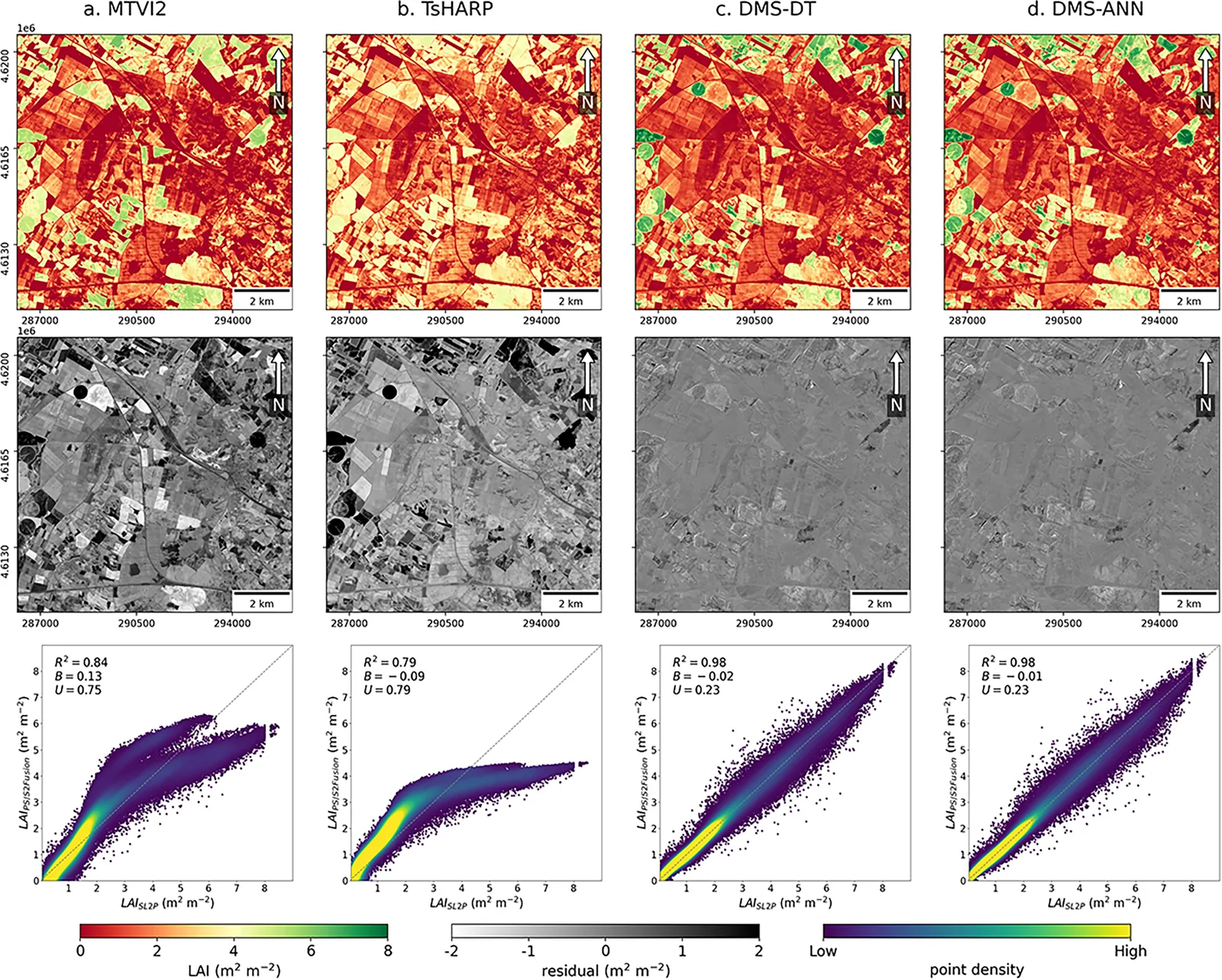
Spatial validation of downscaling methods for LAI using **(a)** MTVI2, **(b)** TsHARP, **(c)** DMS-DT, and **(d)** DMS-ANN. For each method, the downscaled map, residual map (downscaled – Sentinel-2), and scatterplot (yellow indicates higher point density) of Sentinel-2 versus downscaled values with agreement metrics are presented. Results are shown for 9 May 2024.

Residual redistribution was then applied to PlanetScope dates with concurrent Sentinel-2 acquisitions and propagated to independent PlanetScope dates. **Table 1** summarizes the pre- and post-correction metrics across the four study sites and the reference dates. Redistribution consistently lowered uncertainty and raised R^2^ relative to the Sentinel-2 reference, with larger gains for MTVI2/TsHARP than for DMS-DT/DMS-ANN. For fAPAR and FVC, TsHARP and MTVI2 cut uncertainty by 0.03–0.06 and raised R^2^ by 0.06–0.10. For LAI, post-correction errors stayed higher for the vegetation-index methods (uncertainty = 0.43–0.44 m^2^ m^-2^), though R^2^ improved by 0.09–0.19. DMS-DT/DMS-ANN kept the best performance, with uncertainty of 0.11–0.13 m^2^ m^-2^ and R^2^ gains of about 0.02. Following this comparison, all subsequent validation results were obtained using the residual-corrected products.

**Table 1:** Summary of R^2^ and uncertainty values for each variable and downscaling method, calculated over the full downscaled images, before (NoRC) and after (RC) residual correction.

Variable	Method	R^2^		Uncertainty	
		NoRC	RC	NoRC	RC
fAPAR	MTVI	0.90 ± 0.06	0.98 ± 0.04	0.08 ± 0.03	0.02 ± 0.02
	TsHARP	0.91 ± 0.06	0.97 ± 0.04	0.07 ± 0.03	0.04 ± 0.02
	DMS-DT	0.97 ± 0.03	0.98 ± 0.03	0.05 ± 0.02	0.03 ± 0.02
	DMS-ANN	0.97 ± 0.02	0.99 ± 0.02	0.04 ± 0.02	0.03 ± 0.02
FVC	MTVI	0.87 ± 0.07	0.97 ± 0.05	0.09 ± 0.03	0.04 ± 0.03
	TsHARP	0.88 ± 0.07	0.97 ± 0.05	0.09 ± 0.03	0.05 ± 0.02
	DMS-DT	0.95 ± 0.03	0.97 ± 0.05	0.06 ± 0.02	0.04 ± 0.02
	DMS-ANN	0.96 ± 0.01	0.98 ± 0.02	0.06 ± 0.02	0.04 ± 0.01
LAI (m2 m-2)	MTVI	0.81 ± 0.06	0.90 ± 0.06	0.70 ± 0.31	0.26 ± 0.13
	TsHARP	0.76 ± 0.07	0.95 ± 0.06	0.72 ± 0.29	0.29 ± 0.11
	DMS-DT	0.95 ± 0.04	0.97 ± 0.05	0.28 ± 0.15	0.15 ± 0.07
	DMS-ANN	0.96 ± 0.04	0.98 ± 0.01	0.27 ± 0.15	0.16 ± 0.07

Because DMS-DT provided a favorable compromise between predictive performance and computational efficiency, the remaining analysis focuses on this method. **Table 2** summarizes the validation of DMS-DT fusion products after residual redistribution. A total of 9,578 pixel-level observations were evaluated across 35 plots and 35 acquisition dates. Because pixels from the same plot and date are spatially correlated, an additional validation was performed after aggregation to the plot × date level. Across all sites and dates, pixel-level validation yielded R^2^ values of 0.73, 0.67, and 0.75 for fAPAR, FVC, and LAI, respectively. When observations were aggregated at the plot × date level to reduce spatial pseudoreplication, R^2^ increased to 0.80, 0.76, and 0.80, respectively. Uncertainty remained similar or decreased after aggregation, from 0.10 to 0.09 for fAPAR, from 0.13 to 0.10 for FVC, and from 0.75 to 0.60 m^2^ m^-2^ for LAI. Overall, performance remained broadly consistent at the plot × date level. Absolute bias and uncertainty remained low for fAPAR and FVC (|bias|< 0.17; uncertainty< 0.17), whereas LAI showed larger errors (|bias| = 0.18–0.76 m^2^ m^-2^; uncertainty = 0.31–1.23 m^2^ m^-2^), consistent with its wider dynamic range. Herbaceous crops, especially maize, gave the highest R^2^ values, while vineyards were more dispersed, as expected from inter-row reflectance effects, although part of this dispersion may also reflect the uncertainty of the UAV-derived reference data. Within vineyards, cover-crop conditions performed better than tillage, suggesting that inter-row vegetation reduces clumping effects and improves emulation stability. Overall, the fusion strategy produced robust high-resolution biophysical products across contrasting crop types and management conditions. A full comparison of all downscaling methods is given in **Supplementary Table S1** in Supplementary Material.

**Table 2:** Validation statistics for DMS-DT downscaling method applied to the spatio-temporal PlanetScope-Sentinel-2 fusion data, compared against reference data by crop type at pixel and plot per date level.

Crops	Pixel level	*DMS-DT fAPAR*			*DMS-DT FVC*			*DMS-DT LAI*		
	Plot x date level	R^2^	│B│	U	R^2^	│B│	U	R^2^	│B│	U
Alfalfa	48	0.71	0.01	0.12	0.67	0.17	0.15	0.80	0.76	1.02
	9	0.83	0.00	0.10	0.80	0.18	0.12	0.94	0.76	0.80
Maize	88	0.89	0.03	0.12	–	–	–	0.82	0.18	1.02
	16	0.88	0.02	0.12	–	–	–	0.90	0.34	0.74
Winter cereals	90	0.72	0.05	0.14	0.74	0.14	0.17	0.77	0.71	1.23
	13	0.75	0.04	0.13	0.84	0.16	0.14	0.82	0.58	1.09
Vines + cover crop	6847	0.67	0.01	0.08	0.70	0.06	0.13	0.69	0.38	0.31
	8	0.89	0.02	0.05	0.76	0.10	0.09	0.78	0.22	0.24
Vines + tillage	2498	0.61	0.05	0.06	0.57	0.09	0.08	0.68	0.56	0.38
	6	0.64	0.02	0.04	0.64	0.08	0.08	0.78	0.22	0.24
*Overall*	*9578*	*0.73*	*0.03*	*0.10*	*0.67*	*0.11*	*0.13*	*0.75*	*0.52*	*0.75*
	*52*	*0.80*	*0.02*	*0.09*	*0.76*	*0.13*	*0.10*	*0.84*	*0.42*	*0.62*

LAI absolute bias (|B|) and uncertainty (U) values are provided in m^2^ m^-2^ units.

**Figure 6** gives a qualitative comparison of vineyard management patterns in the Raimat (9 May 2024) and Aranyó (14 June 2024) trials. MTVI2/TsHARP produced smoother maps that captured broad gradients but missed fine-scale heterogeneity, whereas DMS-DT/DMS-ANN better preserved local spatial detail.

**Figure 6:**
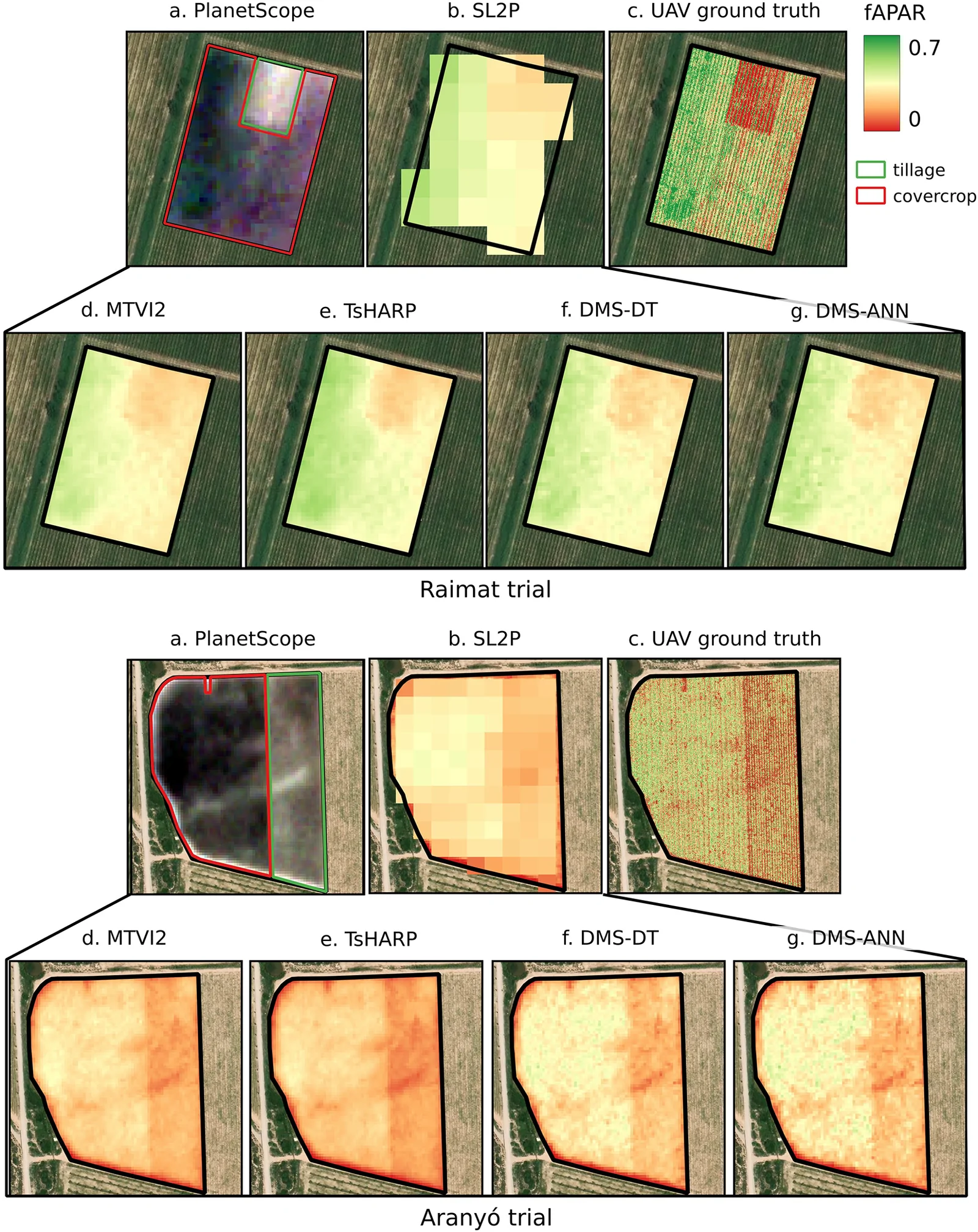
Comparison of fAPAR maps over the Raimat trial (9 May 2024) and the Aranyó trial (14 June 2024) derived from: **(a)** PlanetScope multispectral imagery, **(b)** Sentinel-2 SL2P fAPAR at 20 m resolution, **(c)** UAV-based fAPAR imagery at 0.03 m resolution used as reference data, and **(d–g)** fAPAR maps generated from PS–S2 fusion.

#### 3.2.2 Temporal consistency

To assess temporal consistency, the four downscaling methods were applied to each biophysical variable in the vineyard and maize plots. Field measurements and UAV reference maps were available on only a limited number of dates, so the high-frequency fusion products were evaluated against an a posteriori daily Sentinel-2 SL2P reference series, obtained by temporal interpolation between consecutive Sentinel-2 acquisitions. This comparison assesses consistency with the interpolated Sentinel-2 SL2P series rather than absolute bias, as the fusion models were calibrated using SL2P products. The temporal datasets comprised 121 PlanetScope and 33 Sentinel-2 scenes for the maize plot, 115 PlanetScope and 33 Sentinel-2 scenes for Raimat (vineyard), and 101 PlanetScope and 40 Sentinel-2 scenes for Aranyó (vineyard). The median interval between retained PlanetScope observations was 2 days for maize, 1 day for Raimat, and 1 day for Aranyó, with mean intervals of 3.06, 3.46, and 2.86 days, respectively. However, occasional longer gaps occurred in winter periods, with maximum intervals of 33 days for maize, 41 days for Raimat, and 29 days for Aranyó.

Dates corresponding to harvest or other abrupt vegetation changes detected by PlanetScope but not represented in the interpolated Sentinel-2 series were excluded from the primary temporal-consistency analysis. One date (DOY 298) was excluded in the maize plot, while 11 dates (DOY 258–279) were excluded in the Raimat vineyard, resulting in 12 excluded dates in total as no dates were excluded in Aranyó. Only DMS-DT is presented here because it provided a favorable compromise between predictive performance and computational efficiency; results for the remaining methods appear in **Supplementary Figures S6–S8**. All temporal validation results reported below correspond to residual-corrected products.

The fusion products showed strong temporal consistency across all sites, with low uncertainty relative to the interpolated Sentinel-2 reference series (**Figure 7**). For fAPAR and FVC, uncertainty remained below 0.03 and absolute bias within 0.01, whereas LAI showed higher uncertainty (0.07–0.23 m^2^ m^-2^) and bias ranged from -0.13 to -0.01 m^2^ m^-2^. Including the excluded abrupt-change dates had no effect on maize results and only minor effects in Raimat, changing bias in all variables by 0.01 and uncertainty by up to 0.02 m^2^ m^-2^ in LAI.

**Figure 7:**
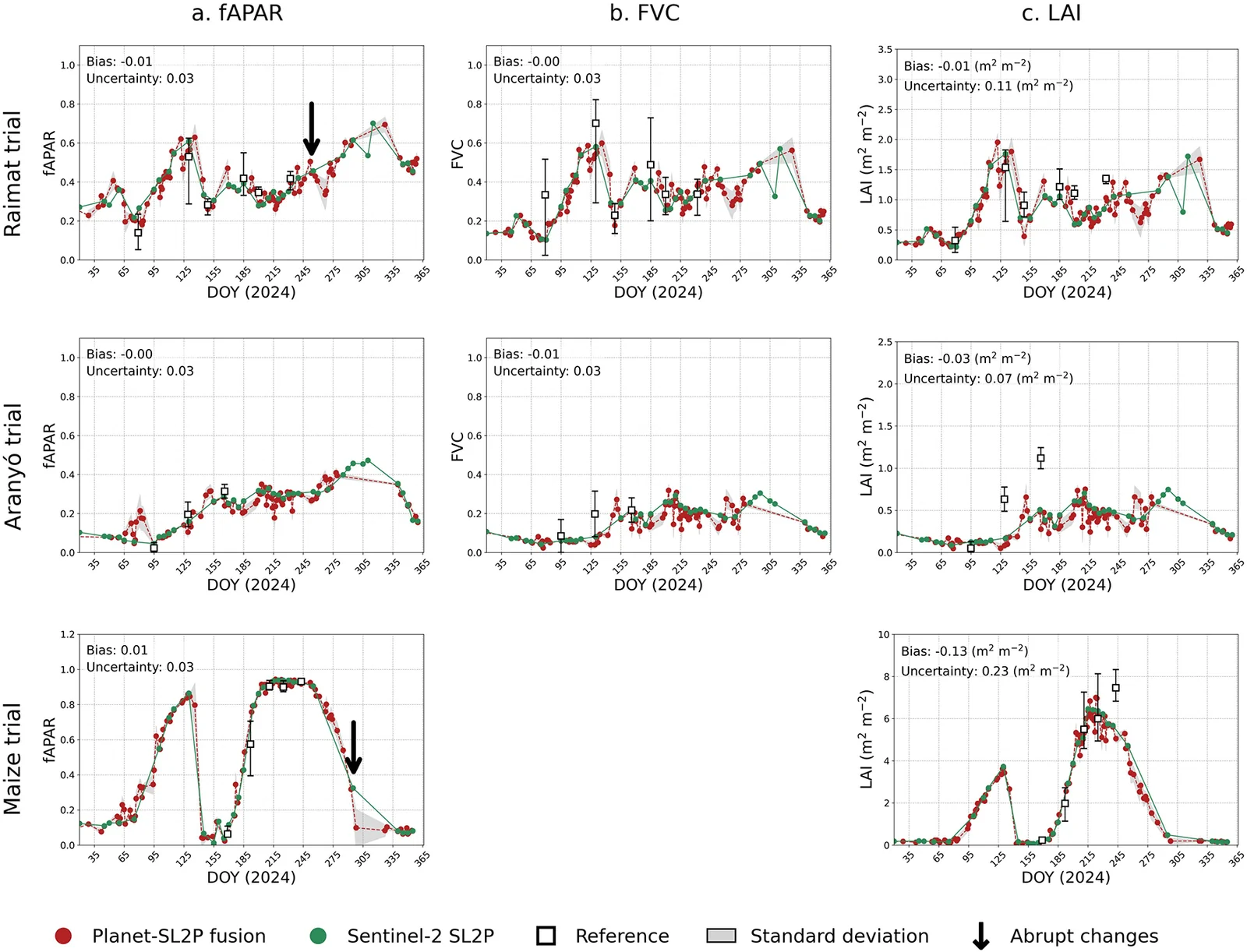
Temporal validation of downscaling methods for **(A)** fAPAR, **(B)** FVC and **(C)** LAI in the Raimat, Aranyó and maize trials. Each panel displays the PlanetScope-Sentinel-2 fusion time series (3 m) using DMS-DT method, the 20 m SL2P benchmark and plot-scale reference data, and reports uncertainties and bias between PlanetScope-SL2P fusion and SL2P. PlanetScope-detected harvests are marked by an arrow in the legend.

#### 3.2.3 Computation resources

Computational cost was benchmarked on a workstation with a 64-bit operating system, 128 GB RAM, and an Intel^®^ Core™ i9–13900 CPU @ 5.6 GHz. MTVI2 and TsHARP were the fastest methods, requiring 44–74 s per variable and trial. DMS-DT was slower, taking 289–405 s, while DMS-ANN was the most time-consuming, at 1,635–1,854 s per variable and trial. Runtimes increased with the number of PlanetScope scenes per trial.

## 4 Discussion

### 4.1 Downscaling biophysical variables

CubeSat constellations such as PlanetScope provide high spatio-temporal resolution imagery, enabling detailed monitoring of vegetation dynamics and short-term events (e.g., harvests; **Figure 7**). However, their limited spectral sampling constrains the direct retrieval of biophysical variables. Previous studies have addressed this limitation either through direct retrieval using radiative transfer and machine-learning approaches (Dong et al., 2024; Johansen et al., 2022) or by downscaling biophysical variables derived from spectrally richer sensors (Houborg and McCabe, 2018a; Sadeh et al., 2021). The present framework follows the downscaling strategy, using Sentinel-2 SL2P products as coarse-resolution references and SuperDove observations to generate 3 m biophysical-variable maps at high-frequency. Compared with previous PlanetScope-based studies, Houborg and McCabe (2018a) and Sadeh et al. (2021) used earlier sensor generations for downscaling, while Johansen et al. (2022) and Dong et al. (2024) used SuperDove for direct biophysical-variable retrieval, relying on a subset of its spectral bands and the LESS radiative transfer model, respectively. In contrast, our approach combines the full available SuperDove spectral information with Sentinel-2 SL2P retrievals based on PROSAIL.

Four downscaling methods were evaluated: two vegetation index-based methods [a simple MTVI2 regression (Sadeh et al., 2021) and a TsHARP-style disaggregation (Agam et al., 2007)] and two Data Mining Sharpener (Gao et al., 2012) machine-learning frameworks (DMS-DT and DMS-ANN). Spatial validation against the Sentinel-2 SL2P reference products showed a clear separation between the two families of methods. For fAPAR and FVC, index-based methods produced larger errors, whereas DMS methods reduced errors substantially, particularly for FVC. LAI was more challenging: index-based methods overestimated intermediate values and underestimated high values, while DMS reduced uncertainty to 0.23–0.29 m^2^·m^-2^. This advantage is particularly relevant for dates without a concurrent Sentinel-2 acquisition, when residual correction must be transferred from a nearby matched date, as DMS methods exploit the full PlanetScope spectral information to capture non-linear, scene-specific relationships such as irrigation-driven variability (Gao et al., 2012).

Because the fusion models were calibrated using SL2P products, agreement with SL2P alone cannot be considered an independent validation. Therefore, the performance of the fused products was assessed against field- and UAV-derived reference data, with 9,578 pixel-level comparisons. Because pixels within the same plot and date are spatially correlated, performance was additionally assessed at the plot × date level, yielding similar or improved performance and confirming that the results were not driven solely by spatial pseudoreplication. All statistics reported below refer to residual-corrected products unless otherwise stated. After residual correction, all methods showed similar agreement with the field- and UAV-derived reference data (**Supplementary Table S1**). LAI remained the most uncertain variable, reflecting the greater difficulty of its downscaling, calibration limitations, and the higher intrinsic uncertainty of SL2P-ANN relative to fAPAR and FVC. Crop-specific patterns were consistent: herbaceous crops showed low bias and higher R^2^, whereas vineyards exhibited larger uncertainty and greater inter-method variability, likely because structural clumping is not fully represented by the SAIL model (Combal et al., 2003). Against the Sentinel-2 SL2P reference, the DMS-DT LAI of alfalfa showed a relative mean absolute difference of 6.9%, slightly above the 4-6% reported by Houborg and McCabe (2018a). Against field- and UAV-derived data, LAI uncertainty was 1.02 m^2^ m^-2^ for both maize and alfalfa and 1.22-1.23 m^2^ m^-2^ for wheat after residual correction. For maize, our uncertainty was higher than the 0.73 m^2^ m^-2^ RMSE reported by Johansen et al. (2022). For winter cereals, it was lower than the 1.73 m^2^ m^-2^ reported by Sadeh et al. (2021), but higher than the 0.80 m^2^ m^-2^ RMSE reported by Dong et al. (2024). The lower errors of Dong et al. (2024) and Johansen et al. (2022) may partly reflect their validation using plot-level or 7 × 7-pixel windows rather than pixel-level assessment. Vineyard performance was also influenced by the larger number of pixels evaluated, mixed soil–cover crop–vine pixels, bidirectional reflectance effect (Tu et al., 2022), and residual errors in UAV-based reference data. Overall, residual correction effectively reduced systematic differences among methods, as also reported by Gao et al. (2012).

For dates without a concurrent Sentinel-2 acquisition, the fused products were compared with an interpolated SL2P reference time series to evaluate temporal consistency rather than absolute bias. Differences remained small for fAPAR and FVC but were larger for LAI, again reflecting the lower robustness of LAI downscaling. DMS-DT was used for the temporal analysis because it combined strong predictive performance with practical processing times (4.8–6.8 min per variable and trial), only slightly slower than MTVI2 or TsHARP and substantially faster than DMS-ANN. This computational advantage supports its use for routine fusion, particularly when frequent processing of multiple variables and acquisition dates is required.

A further strength of the method is that the models were trained in a compact but highly diverse agricultural mosaic, including woody vineyards and herbaceous crops across winter and summer phenological stages. This variability likely improved robustness and temporal generalization within the study region. Rebuilding the model for each PlanetScope–Sentinel-2 match updates the statistical relationship between PlanetScope reflectance and SL2P products according to the prevailing canopy and illumination conditions, thereby limiting temporal drift. Although the PlanetScope harmonization framework incorporates multi-temporal and multi-sensor strategies to minimize radiometric inconsistencies, including potential BRDF effects (Houborg and McCabe, 2018b), it does not explicitly normalize observations to a common viewing geometry. Consequently, residual directional reflectance effects may remain, particularly in row-structured vineyards, and could reduce temporal transferability between successive acquisitions. The applicability of the approach to other regions, crop systems, and observation geometries therefore requires explicit validation.

### 4.2 Limitations and prospects

Several limitations may explain part of the observed bias. For fAPAR, the *R*_∞_ formulation of Baret et al. (1989) was adopted to match the SL2P convention; field values agreed within ±0.02, although assuming a constant *R*_∞_ may add minor crop- and stage-dependent uncertainty (Begue et al., 1991). For FVC, ground-image resolution differed strongly between crop types—0.00026 m px^-^¹ in herbaceous plots versus 0.03 m px^-^¹ in vineyards—limiting the detection of fine cover-crop structures and introducing scale-related uncertainty. LAI required destructive calibration because ceptometer measurements saturate above 5 m^2^·m^-2^ and represent effective rather than true LAI (Jonckheere et al., 2004).

The validation strategy also presents limitations. In herbaceous crops, only three field measurements were used to represent each satellite footprint, which is acceptable for homogeneous canopies (Weiss et al., 2004) but less robust in more variable conditions such as alfalfa. In vineyards, spatial heterogeneity was better captured through UAV campaigns, from which spatially continuous biophysical reference maps were generated using RF-ANN models calibrated against field measurements. These UAV-derived reference maps should not be considered equivalent to direct field observations, as they incorporate uncertainty associated with the empirical models used for spatial extrapolation. Nevertheless, the UAV-based RF-ANN models showed strong performance (R^2^ = 0.83–0.87 for vines; 0.85–0.86 for cover crops; *Appendix B. Vineyard UAV-derived reference data*), although residual modeling errors remain and should be considered when interpreting the validation outcomes. The validation dataset was also unbalanced across crop types, with vineyards contributing substantially more pixel-level comparisons than herbaceous crops. Therefore, pixel-level statistics are more strongly influenced by vineyard observations, and the total of 9,578 comparisons should be interpreted as evaluated pixels rather than independent observations. The plot × date analysis provided a complementary assessment less sensitive to this spatial imbalance.

Another important limitation is that the fusion models were trained on SL2P outputs, which were also used as the benchmark. SL2P is reliable in homogeneous herbaceous canopies but tends to underperform in clumped canopies (Widlowski et al., 2007). fAPAR and FVC results were broadly consistent with the literature (Djamai et al., 2019; Hu et al., 2020), while LAI showed the largest deviations from previous studies (Brown et al., 2021; Djamai et al., 2019; Hu et al., 2020). An increase in absolute bias of approximately 0.13–0.15 m^2^·m^-2^ and a decrease in uncertainty of approximately 0.09–0.35 m^2^·m^-2^ were observed relative to the cited references. The lower LAI uncertainty likely reflects destructive calibration and the presence of SL2P LAI values above 6 m^2^·m^-2^, which reduces apparent saturation. In vineyards, no prior validation studies were available; however, fAPAR and FVC showed higher uncertainty in mixed vine–cover crop pixels, and LAI degraded more strongly with vine density, reflecting the limitations of the SAIL turbid-medium assumptions for clumped canopies (Combal et al., 2003). These discrepancies contrast with the consistent behavior observed in herbaceous crops and align with findings for other clumped canopies such as forests (Brown et al., 2021; Fernandes et al., 2023).

Relative to SL2P, DMS-DT showed a modest decline in performance, especially for LAI. For fAPAR and FVC, R^2^ decreased from 0.80–0.88 with SL2P to 0.67–0.75 with DMS-DT, while absolute bias and uncertainty increased slightly. LAI showed the largest reduction, with R^2^ decreasing from 0.85 to 0.72. Part of this decline is attributable to the evaluation at the 3 m PlanetScope grid, which does not correspond to the native ground sampling distance (3.7–4.2 m) and therefore introduces additional spatial mismatch. In addition, PlanetScope’s multi-satellite constellation may introduce small acquisition-to-acquisition differences in radiometry and viewing geometry, so transferability beyond the present study area and conditions should be tested explicitly. Taken together, these results support the use of DMS-DT when computational efficiency is considered alongside predictive performance. Future work should extend validation to additional crop types and regions, and SL2P itself should also be tested in those settings, since the fusion framework necessarily inherits the strengths and weaknesses of its coarse-resolution reference product. Beyond methodological validation, the resulting high-spatial-resolution, high-frequency biophysical products could provide a potentially useful data source for tracking within-field variability and supporting future applications in irrigation management and canopy monitoring in vineyards and herbaceous crops.

## 5 Conclusions

This study evaluated Sentinel-2 SL2P biophysical products and developed a PlanetScope–Sentinel-2 fusion framework to provide high-resolution maps of vegetation biophysical variables over Mediterranean herbaceous and vineyard crops. SL2P products showed good agreement with reference data for fAPAR and FVC across both crop groups, while LAI was more sensitive to canopy structure and showed a greater tendency towards underestimation in vineyards. These results highlight the challenges of applying coarse-resolution biophysical products to structurally complex woody canopies.

The fusion framework maintained consistency with Sentinel-2 SL2P products while increasing spatial and temporal detail. Among the evaluated approaches, DMS-DT combined competitive predictive performance with substantially lower computational cost than DMS-ANN and was therefore selected for the detailed spatial and temporal analyses. Residual correction improved spatial coherence, while the consistency of the results at the plot × date level indicated that the overall performance was not driven solely by spatial pseudoreplication. The resulting 3 m, high-frequency biophysical maps provide a useful basis for high-resolution vegetation monitoring and could provide a potentially useful data source for applications such as irrigation management, yield prediction, and delineation of within-field management zones, although these applications were not directly evaluated in this study. Beyond direct monitoring, these high-resolution, high-frequency biophysical series could also support the parameterization, calibration, or assimilation of process-based crop models and digital twins, where fine-grained spatial and temporal information may improve the representation of within-field variability. Overall, the framework provides a scalable approach for high-resolution, high-frequency monitoring across contrasting herbaceous and vineyard systems.

## 6 Software availability

The PlanetScope–Sentinel-2 fusion Python code used in this study is openly available at https://github.com/UEA-Irta/PSS2FusionDownscaling (accessed 10 August 2026).

The Python codes used for UAV feature extraction and modeling are available at https://github.com/UEA-Irta/UAVBiophysicalExtraction and https://github.com/UEA-Irta/UAVBiophysicalModelling (accessed 10 August 2026).

## Figures and Tables

**Figure A1:**
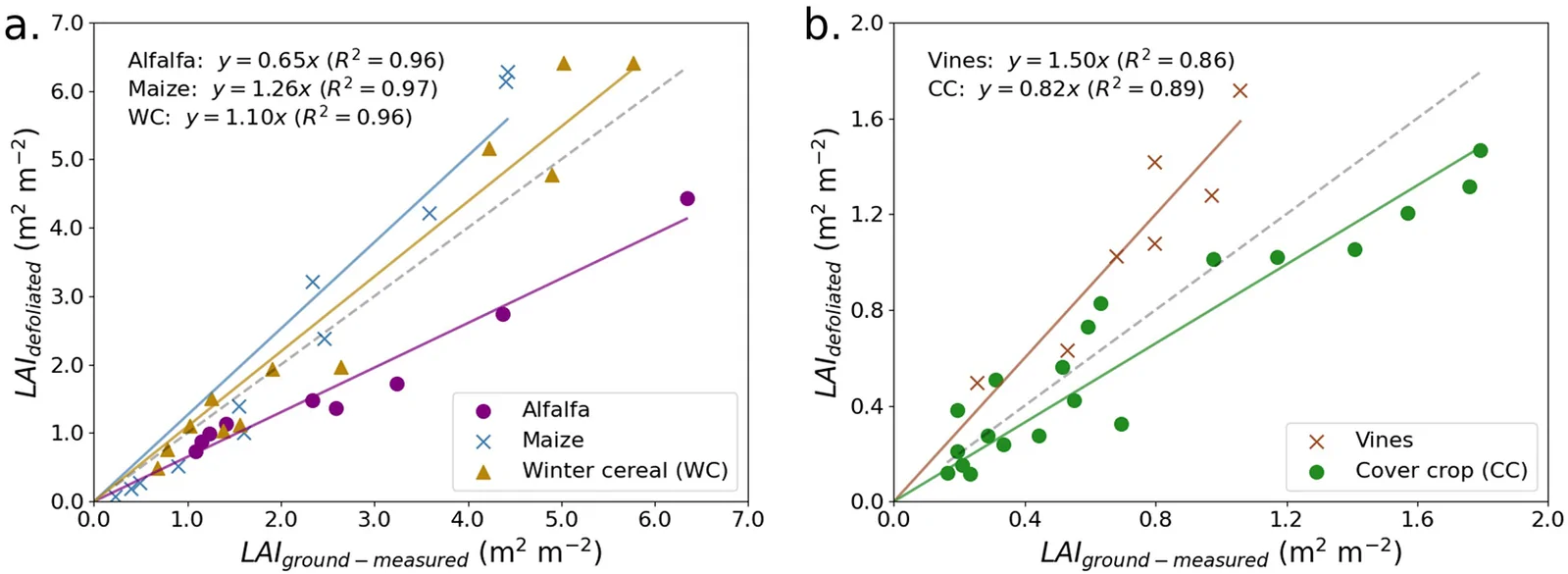
LAI calibration based on defoliated LAI (*LAI*_*defoliated*_) and ceptometer LAI (*LAI*_*ground−measured*_) measurements for **(a)** herbaceous crops, and **(b)** vines with associated cover crops.

**Figure B1:**
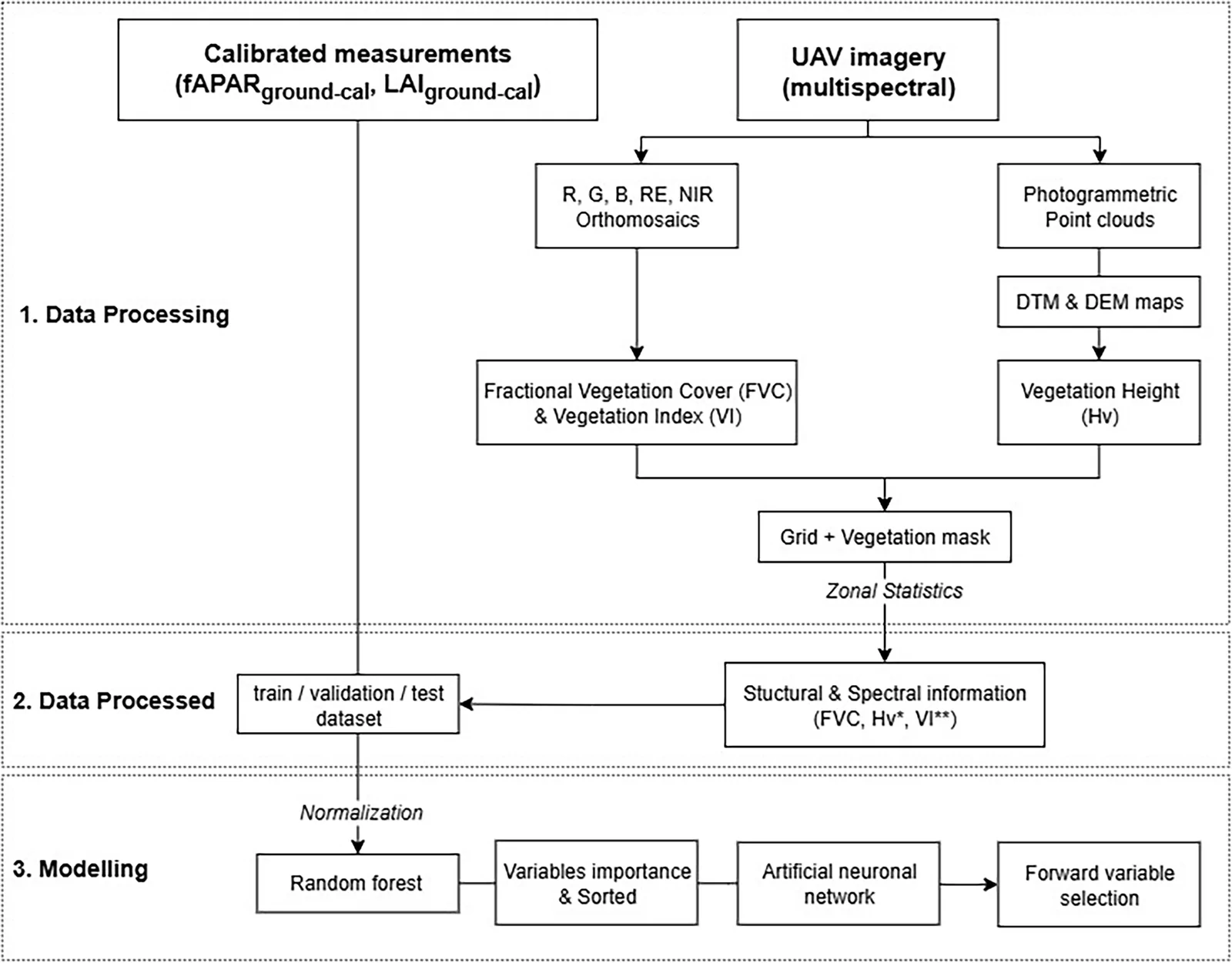
Workflow for estimating vine and cover crop LAI and fAPAR using groundcalibrated data and UAV imagery. The resulting reference LAI and fAPAR datasets are used to validate satellite-derived products. *Five statistics: mean, median, maximum, minimum, and standard deviation.

**Figure B2:**
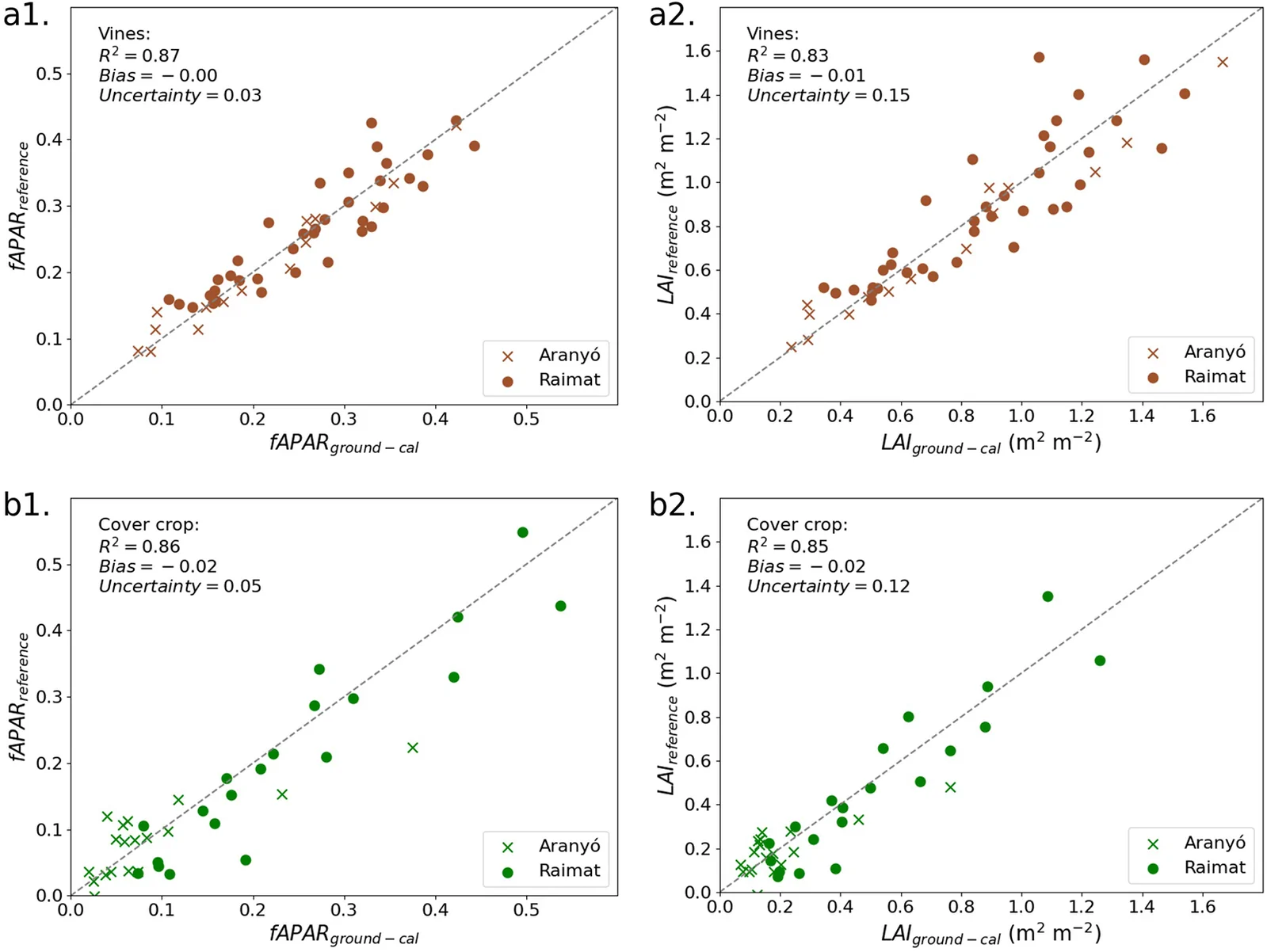
Performance of the RF-ANN UAV models on the test dataset (15%) for vines: **(a1)** fAPAR and **(a2)** LAI; and for cover crops: **(b1)** fAPAR and **(b2)** LAI. The x-axis represents the ground-calibrated data used to train, validation and test the models, while the y-axis shows the corresponding reference data generated by these models, which were subsequently used to validate the satellitederived products.

## Data Availability

The raw data supporting the conclusions of this article will be made available by the authors, without undue reservation.

## Appendix A. fAPAR & LAI calibration

The asymptotic reflectance (R_∞_) for the simplified fAPAR model was obtained from fully closed canopies (FVC ≈ 1). Measured values for different crops were close to the standard reference of 0.94 (Baret et al., 1989), differing by no more than ±0.02. Therefore, R_∞_ = 0.94 was used in all subsequent fAPAR calculations to maintain consistency with previous studies.

The ceptometer-based LAI estimates ( ${\text{LAI}}_{ceptometer}$) were derived from canopy transmittance (*T_C_*), computed as the ratio between below-and above-canopy photosynthetically active radiation (PAR). LAI was obtained by inverting a Beer–Lambert-type formulation following the Norman–Jarvis radiative transfer model (Norman and Jarvis, 1974), which accounts for both direct and diffuse radiation components. The extinction coefficient (K) was parameterized as a function of the solar zenith angle and leaf angle distribution, assuming a spherical distribution (χ = 1) for most crops and a more horizontal distribution (χ = 1.5) for maize (Garrigues et al., 2008). The sunbeam fraction of incoming radiation (*f_b_*) was calculated following Spitters et al. (1986) and leaf absorptivity was fixed at 0.861 (AccuPAR standard mode).

To account for crop-specific biases, zero-intercept linear regressions were calibrated between ceptometer-derived LAI ( ${\text{LAI}}_{ground-measured}$) and destructively measured LAI obtained with a leaf area meter ( ${\text{LAI}}_{defoliated}$) (LI-3100, LI-COR Inc., Lincoln, NE, USA) on a subset of 58 samples. A zero intercept was imposed to avoid non-physical negative LAI values. Although ceptometer saturation has been reported for LAI > 5 m^2^/m^2^ (Jonckheere et al., 2004), no clear saturation was observed in our data, supporting the use of linear regressions. In winter cereals, indirect estimates closely followed the 1:1 line, alfalfa showed overestimation due to its dense homogeneous canopy, whereas maize were underestimated because of canopy clumping and row structure (**Figure A1**). In vineyards, strong underestimation was observed in vine canopies, whereas cover crops exhibited moderate overestimation (**Figure B1**). Crop-specific correction functions were derived and applied to all LAI measurements. These calibrated relationships were subsequently used for the development of UAV-based fAPAR and LAI models described in *Appendix B. Vineyard UAV-derived reference data*.

## Appendix B. Vineyard UAV-derived reference data

Vineyard heterogeneity, both within and between rows, cannot be fully captured by sparse point sampling (Weiss et al., 2004). To overcome this, UAV surveys were conducted to generate very high-resolution biophysical maps for vines and cover crops, supporting satellite product validation. Nine multispectral flights were performed in 2024—six over Raimat (March 19, May 9 and 28, July 3 and 18, August 19) and three over Aranyó (April 4, May 8, June 14)—around solar noon (~14:00 local time) under clear skies. Data were acquired using a Dronehexa XL (DRONETOOLS, Spain) equipped with a MicaSense RedEdge-MX sensor capturing five spectral bands: Blue (455–495 nm), Green (540–580 nm), Red (658–678 nm), Red-Edge (707–727 nm), and NIR (800–880 nm). Flights at 50 m altitude yielded ~0.03 m ground resolution. Radiometric, geometric, and atmospheric corrections were applied using Spectralon-based field calibration (FieldSpec 4 spectroradiometer) and RTK-GNSS ground control (ZED-F9P, ArduSimple). Mosaics and point-cloud products—including the digital surface model (DSM) and digital terrain model (DTM)—were generated in Agisoft Metashape, with final edits in QGIS 3.36. The vegetation height model was computed as the difference between the DSM and DTM. SAVI and vegetation-height (*H_V_*) thresholds were determined using the Otsu automatic thresholding method (Otsu, 1979) and verified by visual inspection.

A total of 580 ground sampling units (332 vine, 248 cover crop) were used to build four empirical models—one per variable (fAPAR, LAI) and vegetation type (vine, cover crop). Input features included FVC, *H_V_*, and UAV-derived vegetation indices (NDVI, NDWI, CIg, SAVI, MTVI2, NDRE, VARI, Iron Oxide Index, NGRDI), summarized by mean, median, maximum, minimum, and standard deviation. A two-step RF–ANN approach was applied: Random Forest ranked variables by importance, followed by forward selection to define the optimal subset for training an Artificial Neural Network (one hidden layer, 85 neurons, ReLU activation, Adam optimizer). Data were split 70/15/15% for training, validation, and testing, with normalization and early stopping to avoid overfitting. The complete workflow is summarized in **Figure B1**.

Models showed high bias despite canopy differences. For vines, key predictors were FVC, $H_{V_{mean}}$, $NDRE_{50th},\;\;SAVI_{25th}$ and $NDWI_{50th}$, achieving a R^2^ of 0.87 and uncertainty of 0.03 for fAPAR and R^2^ of 0.83 and uncertainty of 0.15 m^2^ m^-2^ for LAI on the test dataset. For cover crops, the main predictors were FVC, $MTVI2_{50th},\;\;VARI_{25th},$, $SAVI_{50th}$, $NDVI_{50th}$ and $H_{V_{mean}}$ with an R^2^ of 0.86 and uncertainty of 0.05 for fAPAR and R^2^ = 0.85 and uncertainty = 0.12 m^2^ m^-2^ for LAI. The results are shown in **Figure B2**.

**Table:** Variable nomenclature.

Variable nomenclature	
*fAPAR*	Fraction of absorbed photosynthetically active radiation
*FVC*	Fraction of vegetation cover
*LAI*	Leaf area index (m^2^/m^2^)
*fAPAR/LAI _ground-measured_*	Biophysical variables measured with the ceptometer
*LAI_defoliated_*	LAI defoliation measured with the LI-3100, used for LAI calibration
*fAPAR/LAI _ground-cal_*	Calibrated biophysical variables
*fAPAR/FVC/LAI _reference_*	Biophysical variables used as reference data
*fAPAR/FVC/LAI _SL2P_*	Biophysical variable retrievals from Sentinel-2 SL2P
*fAPAR/FVC/LAI _Planet-SL2P fusion_*	Biophysical variable emulations from PlanetScope-Sentinel-2 SL2P fusion
Methods nomenclature	
*SL2P*	Sentinel-2 Simplified Level-2 Prototype processor
*MTVI2*	Fusion method using a linear regression with the modified triangular vegetation index 2
*TsHARP*	Fusion method using an algorithm for sharpening thermal imagery (TsHARP)
*DMS-DT*	Fusion method using the Data Mining Sharpener with a bagged ensemble of decision trees
*DMS-ANN*	Fusion method using the Data Mining Sharpener with an artificial neural network
Statistics nomenclature	
*R^2^*	The coefficient of determination
*B*	Bias (bias)
*|B|*	Absolut bias (|bias|)
*P*	Precision
*U*	Uncertainty (RMSE)
*UAR*	Uncertainty agreement ratio
