# Supplementary material for: Validation and high-resolution downscaling of Sentinel-2 biophysical variables using PlanetScope in vineyard and annual cropping systems
Source: Front Plant Sci. 2026 Oct 30;17:1974500. doi: 10.3389/fpls.2026.1974500 (PMC13760455; doi:10.3389/fpls.2026.1974500)
Supplement: Supplementary file 1 [file DataSheet1.pdf]

Supplementary Materials for:

Validation of Sentinel-2 SL2P and Fused PlanetScope/Sentinel-2 Biophysical Variables in Agricultural Fields

Authors: César Minuesa [a], Jordi Gené-Mola [a], Elia Capdevila [a], Joaquim Bellvert [a]*

Affiliations: [a] IRTA, Efficient Use of Water in Agriculture, Fruitcentre, Lleida, Catalonia, Spain

**Corresponding author. E-mail address: cesar.minuesa@irta.cat*

The file includes:

Fig. S1. Paper side for spatial performance of downscaling methods for fAPAR using (a) MTVI2, (b) TsHARP, (c) DMS-DT, and (d) DMS-ANN. For each method, the downscaled map, residual map (downscaled – Sentinel-2), and scatterplot of Sentinel-2 versus downscaled values with agreement metrics are presented.

Fig. S2. Paper side for spatial performance of downscaling methods for FVC using (a) MTVI2, (b) TsHARP, (c) DMS-DT, and (d) DMS-ANN. For each method, the downscaled map, residual map (downscaled – Sentinel-2), and scatterplot of Sentinel-2 versus downscaled values with agreement metrics are presented.

Fig. S3. Supplementary side for spatial performance of downscaling methods for fAPAR using (a) MTVI2, (b) TsHARP, (c) DMS-DT, and (d) DMS-ANN. For each method, the downscaled map, residual map (downscaled – Sentinel-2), and scatterplot of Sentinel-2 versus downscaled values with agreement metrics are presented.

Fig. S4. Supplementary side for spatial performance of downscaling methods for FVC using (a) MTVI2, (b) TsHARP, (c) DMS-DT, and (d) DMS-ANN. For each method, the downscaled map, residual map (downscaled – Sentinel-2), and scatterplot of Sentinel-2 versus downscaled values with agreement metrics are presented.

Fig. S5. Supplementary side for spatial performance of downscaling methods for LAI using (a) MTVI2, (b) TsHARP, (c) DMS-DT, and (d) DMS-ANN. For each method, the downscaled map, residual map (downscaled – Sentinel-2), and scatterplot of Sentinel-2 versus downscaled values with agreement metrics are presented.

Table S1. Spatial validation statistics for downscaling methods applied to the PlanetScope-Sentinel-2 fusion data, compared against ground truth data at pixel level.

Fig. S6. Temporal validation of downscaling methods for (a) fAPAR and (b) LAI in the maize trial. Results are shown by downscaling method in rows (MTVI2, TsHARP, DMS-DT, DMS-ANN). Each panel displays the PS-S2 fusion time series (3 m), the 20 m SL2P benchmark and plot-scale ground truth data, and reports uncertainties for SL2P–ground, PS-S2 fusion–ground and PS-S2 fusion–SL2P. PlanetScope-detected harvests are marked by an arrow in the legend; dates detected only by PlanetScope were excluded from PS-S2 fusion–SL2P uncertainty calculations.

Fig. S7. Temporal validation of downscaling methods for (a) fAPAR, (b) FVC and (c) LAI in the Raimat trial (vineyard). Results are shown by downscaling method in rows (MTVI2, TsHARP, DMS-DT, DMS-ANN). Each panel displays the PS-S2 fusion time series at 3 m, the 20 m SL2P benchmark and plot-scale in situ measurements, and reports uncertainties for SL2P–ground, PS-S2 fusion–ground and PS-S2 fusion–SL2P. PlanetScope-detected harvests are indicated by an arrow in the legend, and dates detected only by PlanetScope were excluded from PS-S2 fusion–SL2P uncertainty calculations.

Fig. S8. Temporal validation of downscaling methods for (a) fAPAR, (b) FVC and (c) LAI in the Aranyo trial (vineyard). Results are shown by downscaling method in rows (MTVI2, TsHARP, DMS-DT, DMS-ANN). Each panel displays the PS-S2 fusion time

series at 3 m, the 20 m SL2P benchmark and plot-scale in situ measurements, and reports uncertainties for SL2P–ground, PS-S2 fusion–ground and PS-S2 fusion–SL2P.

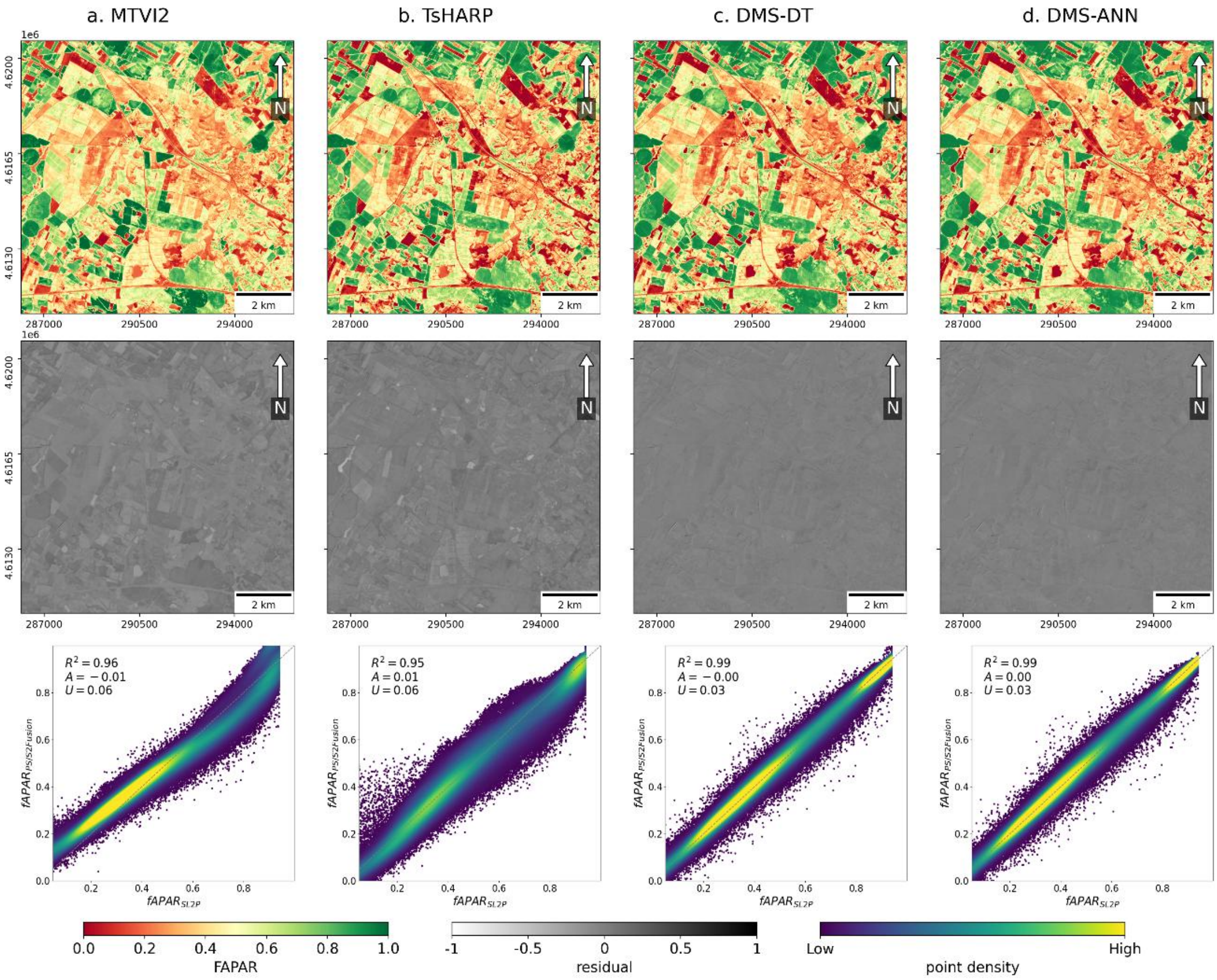

Fig. S1. Paper side for spatial performance of downscaling methods for fAPAR using (a) MTVI2, (b) TsHARP, (c) DMS-DT, and (d) DMS-ANN. For each method, the downscaled map, residual map (downscaled – Sentinel-2), and scatterplot of Sentinel-2 versus downscaled values with agreement metrics are presented.

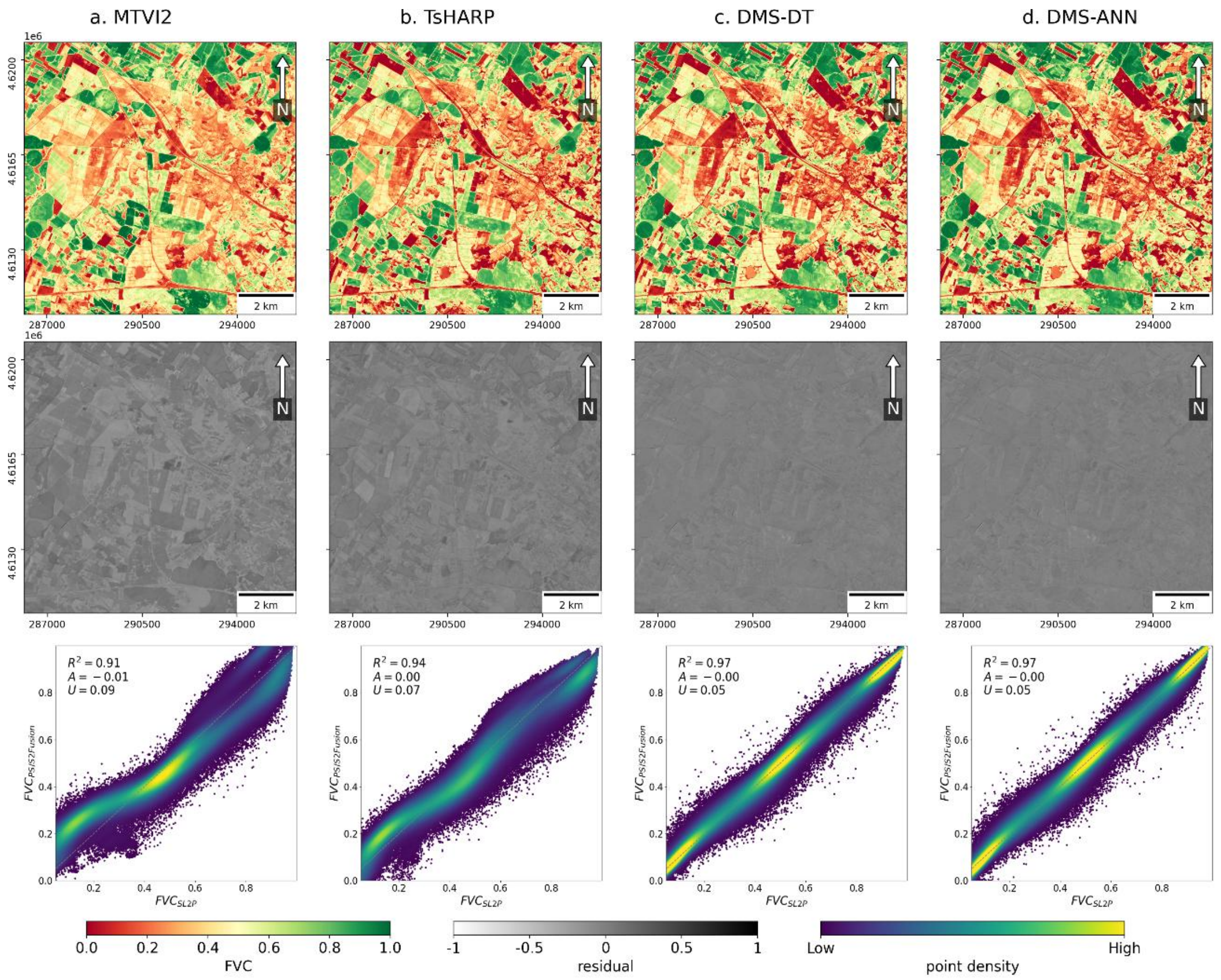

Fig. S2. Paper side for spatial performance of downscaling methods for FVC using (a) MTVI2, (b) TsHARP, (c) DMS-DT, and (d) DMS-ANN. For each method, the downscaled map, residual map (downscaled – Sentinel-2), and scatterplot of Sentinel-2 versus downscaled values with agreement metrics are presented.

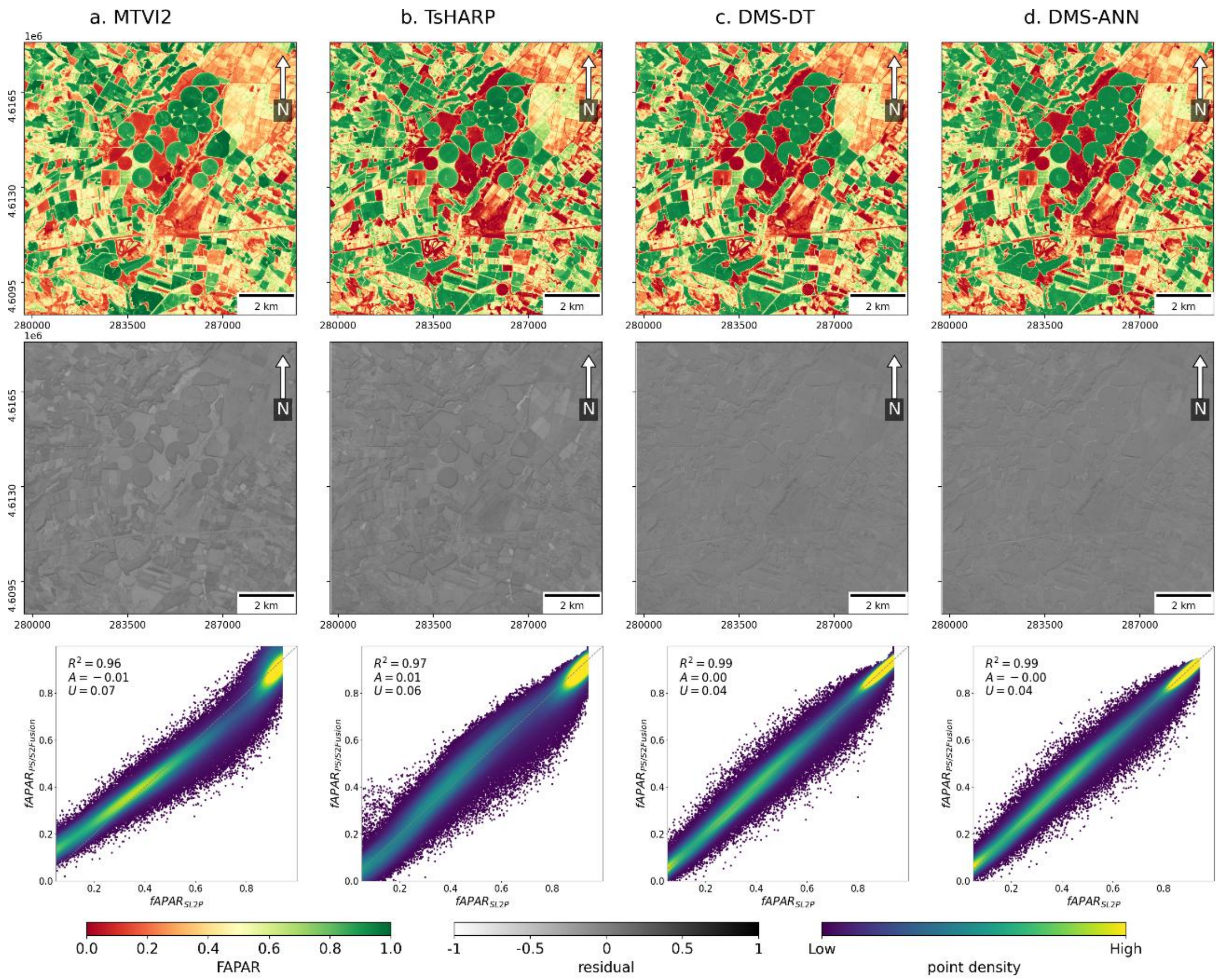

Fig. S3. Supplementary side for spatial performance of downscaling methods for fAPAR using (a) MTVI2, (b) TsHARP, (c) DMS-DT, and (d) DMS-ANN. For each method, the downscaled map, residual map (downscaled – Sentinel-2), and scatterplot of Sentinel-2 versus downscaled values with agreement metrics are presented.

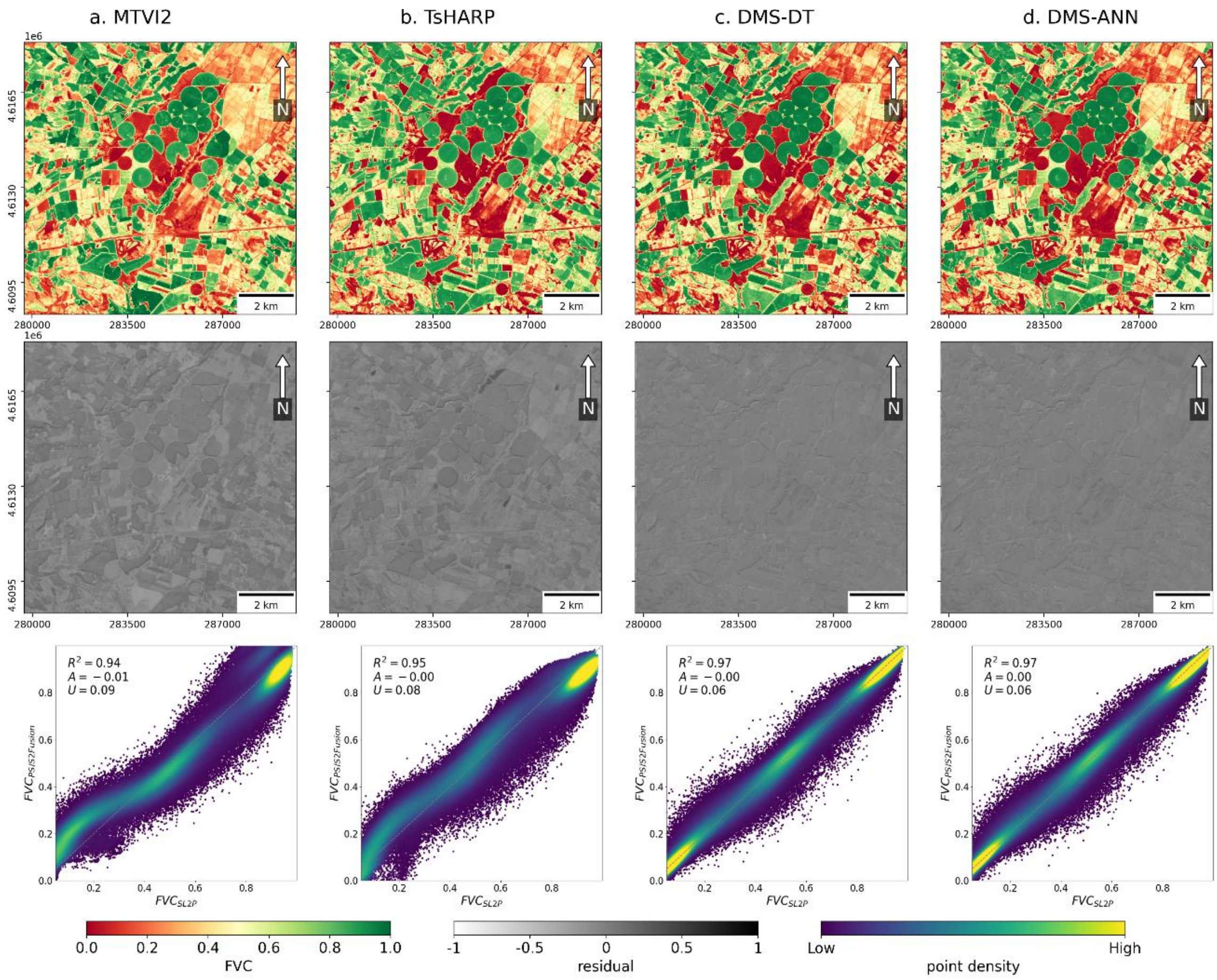

Fig. S4. Supplementary side for spatial performance of downscaling methods for FVC using (a) MTVI2, (b) TsHARP, (c) DMS-DT, and (d) DMS-ANN. For each method, the downscaled map, residual map (downscaled – Sentinel-2), and scatterplot of Sentinel-2 versus downscaled values with agreement metrics are presented.

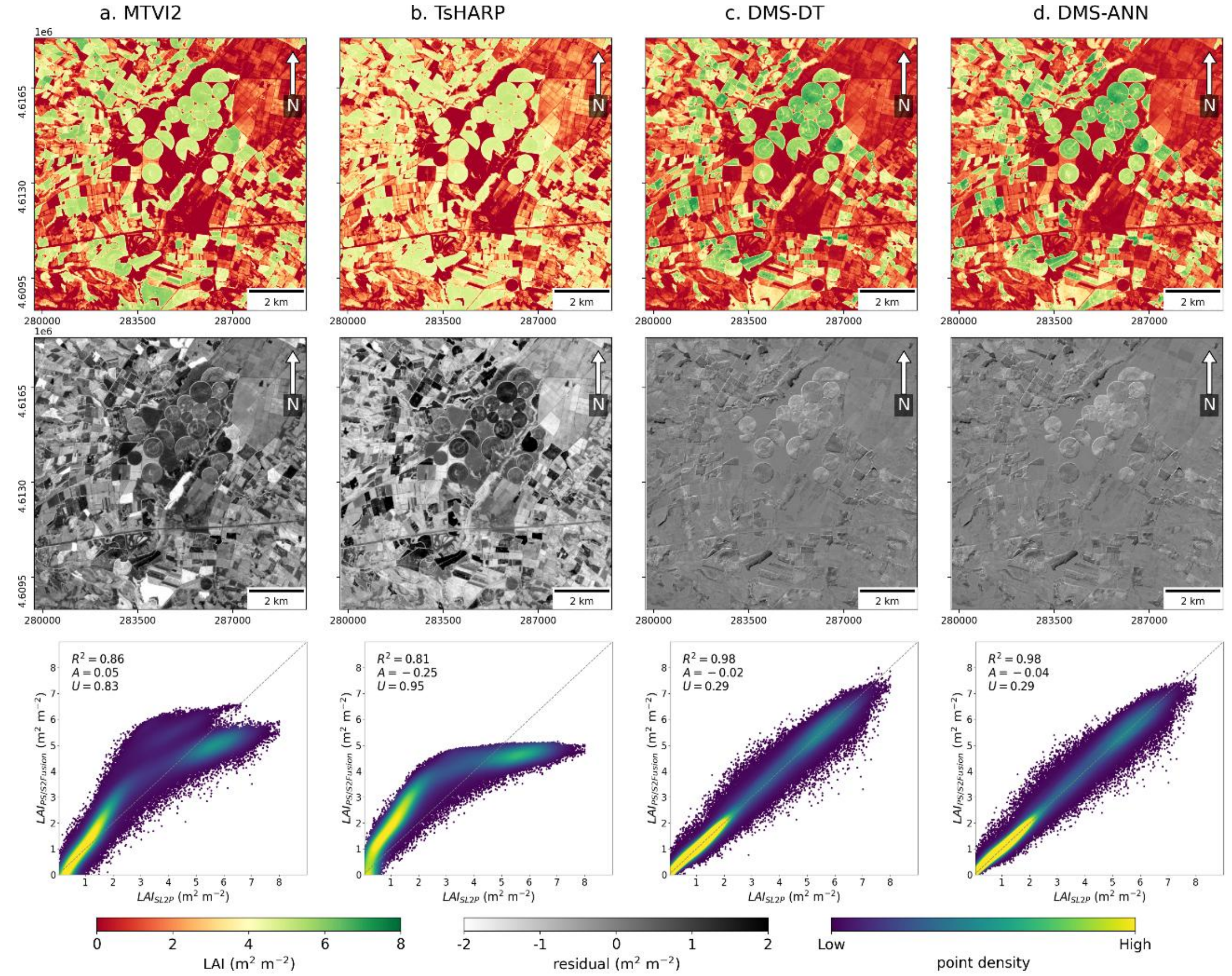

Fig. S5. Supplementary side for spatial performance of downscaling methods for LAI using (a) MTVI2, (b) TsHARP, (c) DMS-DT, and (d) DMS-ANN. For each method, the downscaled map, residual map (downscaled – Sentinel-2), and scatterplot of Sentinel-2 versus downscaled values with agreement metrics are presented.

Table S1. Spatial validation statistics for downscaling methods applied to the PlanetScope-Sentinel-2 fusion data, compared against ground truth data at pixel level.

**Crops**	**N**	**R^2**	**$\lvert A \rvert$**	**P**	**U**	**UAR**	**R^2**	**$\lvert A \rvert$**	**P**	**U**	**UAR**	**R^2**	**$\lvert A \rvert$**	**P**	**U**	**UAR**
		MTVI2 fAPAR					*MTVI2 FVC*					*MTVI2 LAI*				
Alfalfa	48	0.72	0.01	0.12	0.12	0.50	0.66	0.16	0.22	0.15	0.31	0.79	0.82	1.31	1.03	0.46
Maize	88	0.89	0.03	0.12	0.12	0.51	-	-	-	-	-	0.81	0.23	1.01	0.99	0.57
Winter cereals	90	0.73	0.05	0.15	0.14	0.49	0.74	0.14	0.22	0.17	0.49	0.76	0.71	1.41	1.22	0.46
Vines + covercrop	6847	0.69	0.02	0.08	0.08	0.49	0.70	0.05	0.14	0.13	0.35	0.60	0.30	0.44	0.32	0.65
Vines + tillage	2498	0.59	0.04	0.08	0.07	0.48	0.56	0.09	0.13	0.09	0.30	0.54	1.17	1.38	0.50	0.24
Overall	*9578*	*0.72*	*0.03*	*0.11*	*0.11*	*0.49*	*0.66*	*0.11*	*0.18*	*0.13*	*0.36*	*0.70*	*0.65*	*1.11*	*0.81*	*0.47*
		TsHARP fAPAR					*TsHARP FVC*					*TsHARP LAI*				
Alfalfa	48	0.72	0.01	0.12	0.12	0.46	0.66	0.16	0.22	0.15	0.31	0.80	0.82	1.32	1.03	0.46
Maize	88	0.89	0.02	0.12	0.11	0.52	-	-	-	-	-	0.83	0.20	0.98	0.96	0.60
Winter cereals	90	0.73	0.05	0.15	0.14	0.48	0.74	0.14	0.22	0.16	0.49	0.77	0.71	1.41	1.22	0.49
Vines + covercrop	6847	0.70	0.02	0.08	0.08	0.49	0.70	0.05	0.14	0.13	0.35	0.58	0.35	0.49	0.25	0.64
Vines + tillage	2498	0.60	0.04	0.08	0.07	0.49	0.56	0.09	0.13	0.09	0.35	0.45	0.62	0.67	0.34	0.33
Overall	*9578*	*0.73*	*0.03*	*0.11*	*0.11*	*0.49*	*0.67*	*0.11*	*0.18*	*0.13*	*0.38*	*0.68*	*0.54*	*0.97*	*0.76*	*0.50*
		DMS-DT fAPAR					*DMS-DT FVC*					*DMS-DT LAI*				
Alfalfa	48	0.71	0.01	0.12	0.12	0.50	0.67	0.17	0.23	0.15	0.29	0.80	0.76	1.29	1.05	0.42
Maize	88	0.89	0.03	0.12	0.12	0.52	-	-	-	-	-	0.82	0.18	1.04	1.02	0.60
Winter cereals	90	0.72	0.05	0.15	0.14	0.49	0.74	0.14	0.22	0.17	0.47	0.77	0.71	1.41	1.23	0.46
Vines + covercrop	6847	0.67	0.01	0.08	0.08	0.48	0.70	0.06	0.14	0.13	0.33	0.69	0.38	0.40	0.31	0.60
Vines + tillage	2498	0.61	0.05	0.08	0.06	0.51	0.57	0.09	0.13	0.08	0.39	0.68	0.56	0.65	0.38	0.50
Overall	*9578*	*0.73*	*0.03*	*0.11*	*0.10*	*0.50*	*0.67*	*0.11*	*0.18*	*0.13*	*0.38*	*0.75*	*0.52*	*0.96*	*0.75*	*0.52*
		DMS-ANN fAPAR					*DMS-ANN FVC*					*DMS-ANN LAI*				
Alfalfa	48	0.70	0.01	0.12	0.12	0.50	0.66	0.16	0.22	0.12	0.50	0.80	0.82	1.31	1.03	0.46
Maize	88	0.89	0.03	0.12	0.12	0.52	-	-	-	-	-	0.82	0.23	1.01	0.99	0.57
Winter cereals	90	0.73	0.05	0.15	0.14	0.49	0.74	0.14	0.22	0.17	0.49	0.77	0.71	1.41	1.22	0.46
Vines + covercrop	6847	0.69	0.02	0.08	0.07	0.49	0.70	0.05	0.14	0.13	0.35	0.63	0.28	0.71	0.22	0.73
Vines + tillage	2498	0.59	0.04	0.08	0.08	0.48	0.56	0.09	0.13	0.09	0.30	0.61	0.67	0.42	0.30	0.19
Overall	*9578*	*0.72*	*0.03*	*0.11*	*0.11*	*0.50*	*0.67*	*0.11*	*0.18*	*0.13*	*0.41*	*0.73*	*0.54*	*0.97*	*0.75*	*0.48*

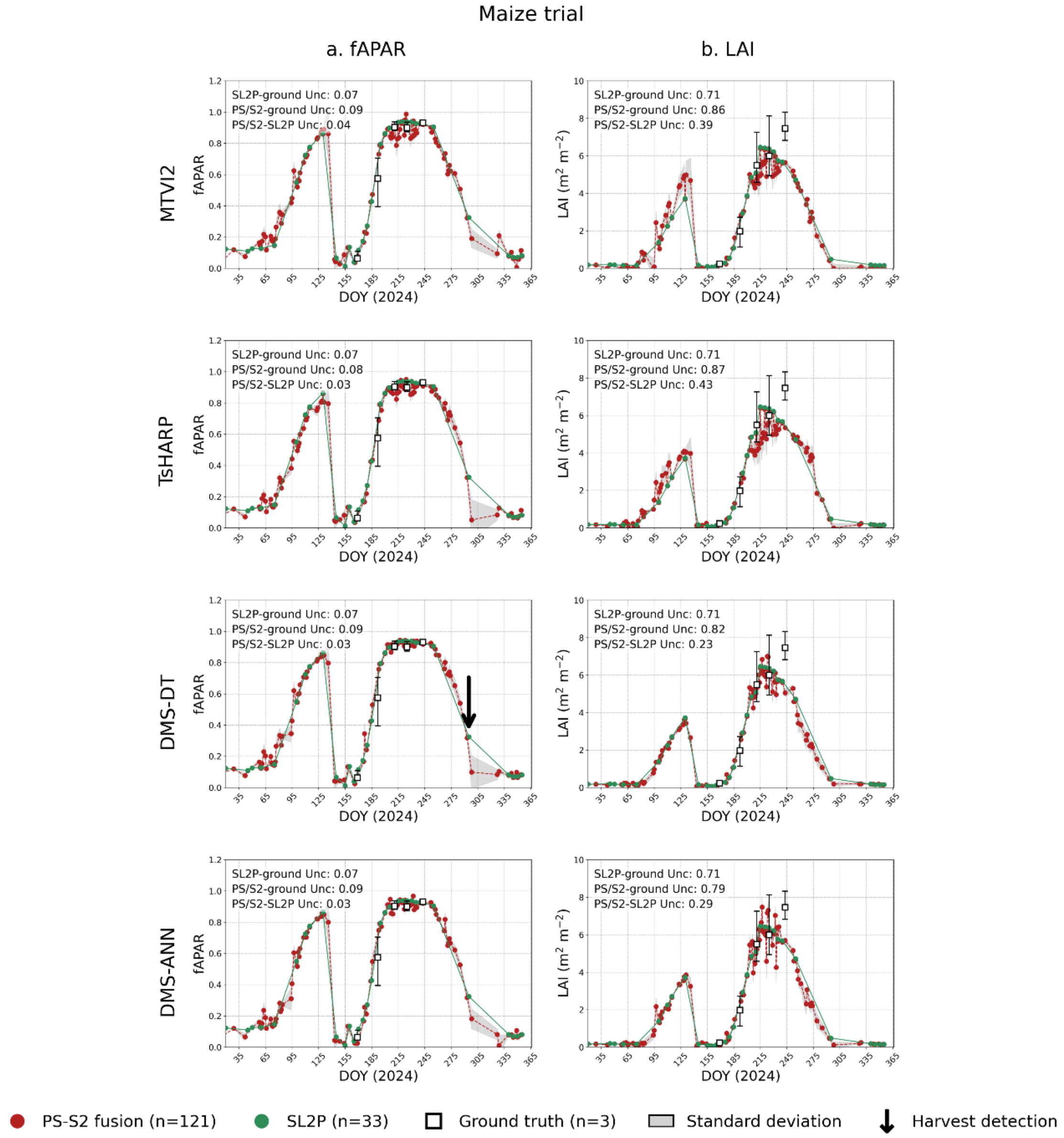

Fig. S6. Temporal validation of downscaling methods for (a) fAPAR and (b) LAI in the maize trial. Results are shown by downscaling method in rows (MTVI2, TsHARP, DMS-DT, DMS-ANN). Each panel displays the PS-S2 fusion time series (3 m), the 20 m SL2P benchmark and plot-scale ground truth data, and reports uncertainties for SL2P–ground, PS-S2 fusion–ground and PS-S2 fusion–SL2P. PlanetScope-detected harvests are marked by an arrow in the legend; dates detected only by PlanetScope were excluded from PS-S2 fusion–SL2P uncertainty calculations.

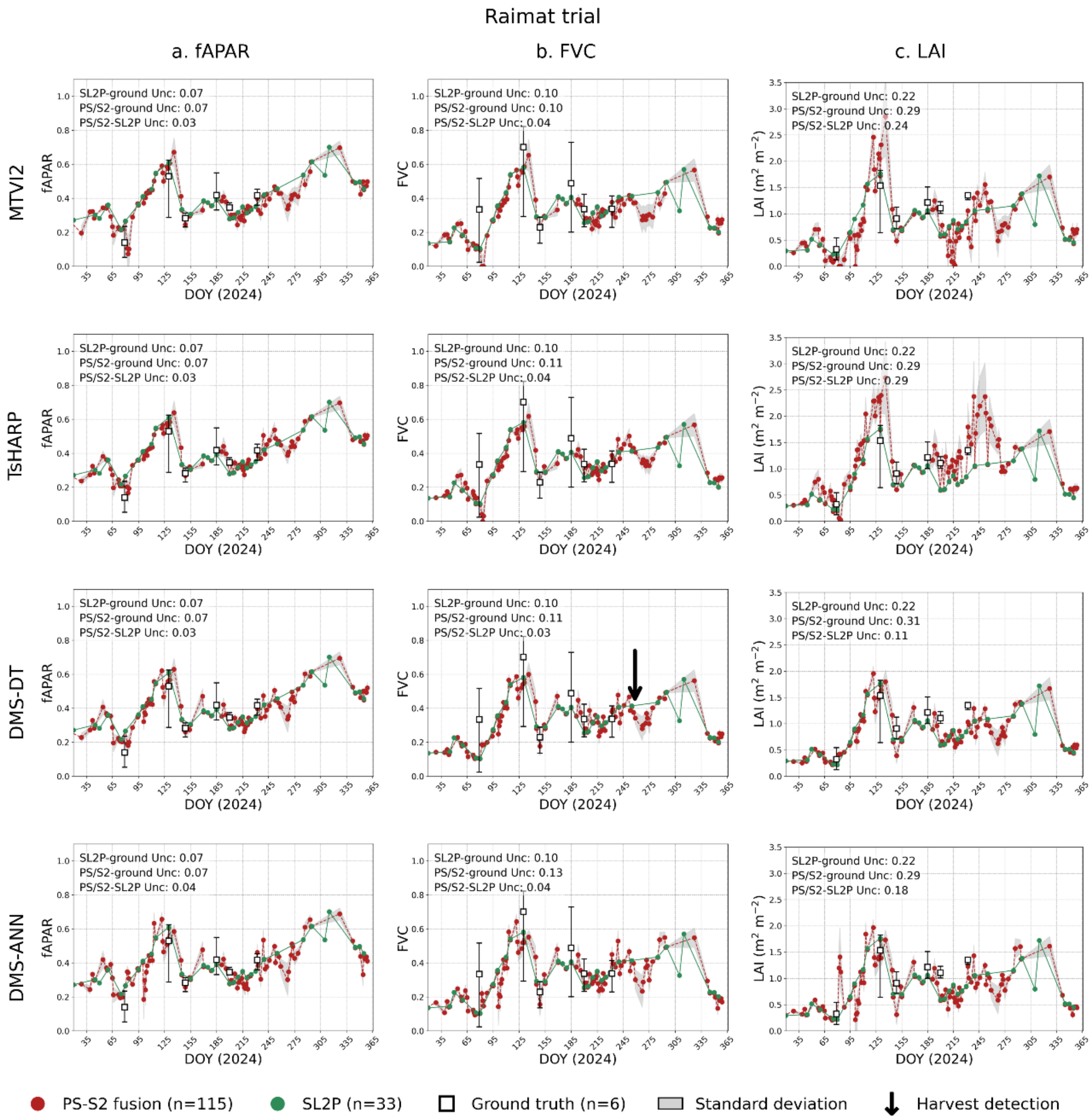

Fig. S7. Temporal validation of downscaling methods for (a) fAPAR, (b) FVC and (c) LAI in the Raimat trial (vineyard). Results are shown by downscaling method in rows (MTVI2, TsHARP, DMS-DT, DMS-ANN). Each panel displays the PS-S2 fusion time series at 3 m, the 20 m SL2P benchmark and plot-scale in situ measurements, and reports uncertainties for SL2P–ground, PS-S2 fusion–ground and PS-S2 fusion–SL2P. PlanetScope-detected harvests are indicated by an arrow in the legend, and dates detected only by PlanetScope were excluded from PS-S2 fusion–SL2P uncertainty calculations.

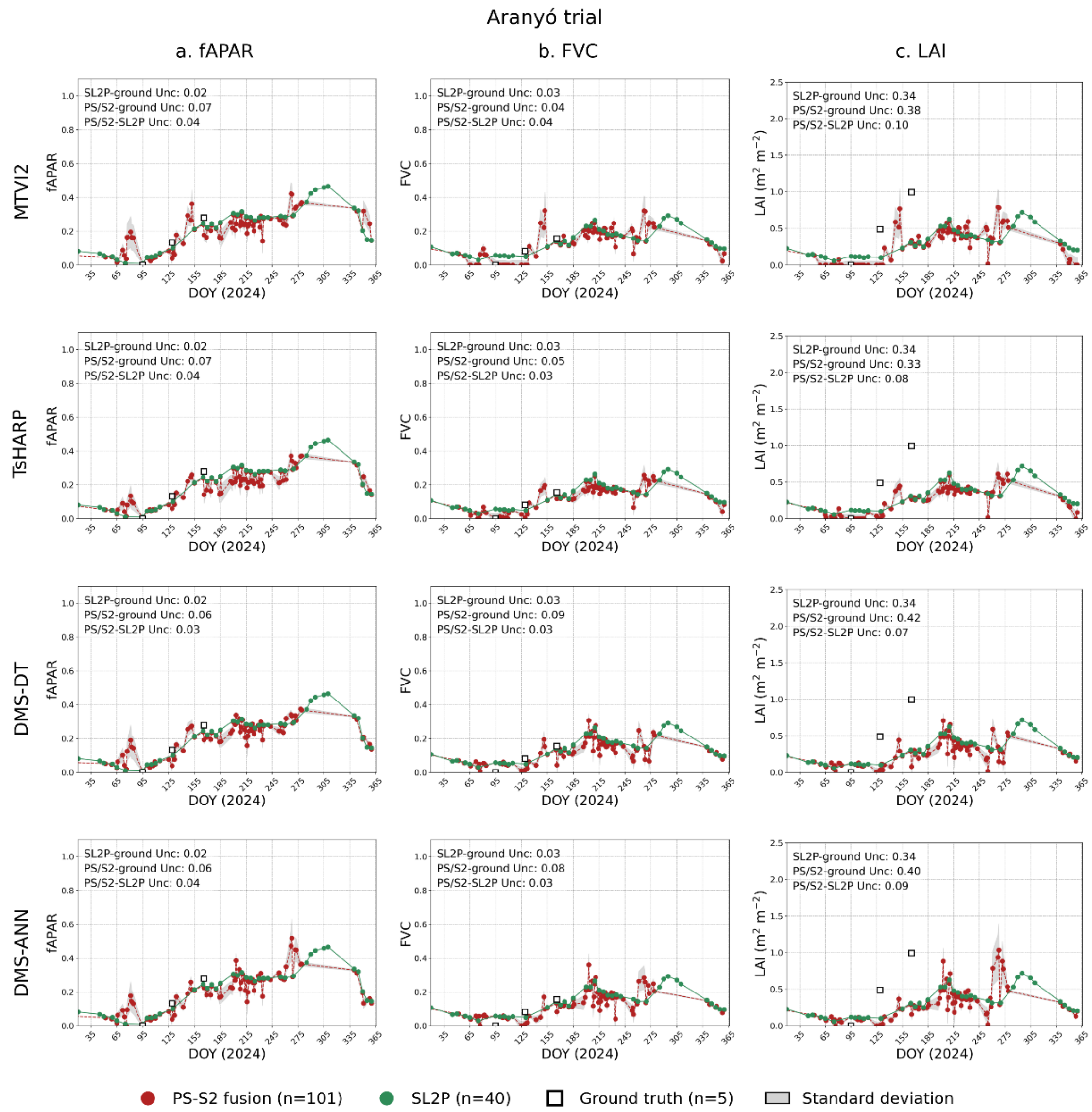

Fig. S8. Temporal validation of downscaling methods for (a) fAPAR, (b) FVC and (c) LAI in the Aranyo trial (vineyard). Results are shown by downscaling method in rows (MTVI2, TsHARP, DMS-DT, DMS-ANN). Each panel displays the PS-S2 fusion time series at 3 m, the 20 m SL2P benchmark and plot-scale in situ measurements, and reports uncertainties for SL2P–ground, PS-S2 fusion–ground and PS-S2 fusion–SL2P.
